# Heart Rate Variability Reveals Altered Autonomic Regulation in Response to Myocardial Infarction in Experimental Animals

**DOI:** 10.3389/fcvm.2022.843144

**Published:** 2022-05-02

**Authors:** Emanuele Pizzo, Silvia Berrettoni, Ridhima Kaul, Daniel O. Cervantes, Valeria Di Stefano, Sudhir Jain, Jason T. Jacobson, Marcello Rota

**Affiliations:** ^1^Department of Physiology, New York Medical College, Valhalla, NY, United States; ^2^Department of Pathology, Microbiology and Immunology, New York Medical College, Valhalla, NY, United States; ^3^Department of Cardiology, Westchester Medical Center, Valhalla, NY, United States

**Keywords:** heart rate variability (HRV), myocardial infarction, autonomic regulation of heart, mouse, electrocardiogram

## Abstract

The analysis of beating rate provides information on the modulatory action of the autonomic nervous system on the heart, which mediates adjustments of cardiac function to meet hemodynamic requirements. In patients with myocardial infarction, alterations of heart rate variability (HRV) have been correlated to the occurrence of arrhythmic events and all-cause mortality. In the current study, we tested whether experimental rodent models of myocardial infarction recapitulate dynamics of heart rate variability observed in humans, and constitute valid platforms for understanding mechanisms linking autonomic function to the development and manifestation of cardiovascular conditions. For this purpose, HRV was evaluated in two engineered mouse lines using electrocardiograms collected in the conscious, restrained state, using a tunnel device. Measurements were obtained in naïve mice and animals at 3–∼28 days following myocardial infarction, induced by permanent coronary artery ligation. Two mouse lines with inbred and hybrid genetic background and, respectively, homozygous (Homo) and heterozygous (Het) for the MerCreMer transgene, were employed. In the naïve state, Het female and male mice presented prolonged RR interval duration (∼9%) and a ∼4-fold increased short- and long-term RR interval variability, with respect to sex-matched Homo mice. These differences were abrogated by pharmacological interventions inhibiting the sympathetic and parasympathetic axes. At 3–∼14 days after myocardial infarction, RR interval duration increased in Homo mice, but was not affected in Het animals. In contrast, Homo mice had minor modifications in HRV parameters, whereas substantial (> 50%) reduction of short- and long-term RR interval variation occurred in Het mice. Interestingly, *ex vivo* studies in isolated organs documented that intrinsic RR interval duration increased in infarcted vs. non-infarcted Homo and Het hearts, whereas RR interval variation was not affected. In conclusion, our study documents that, as observed in humans, myocardial infarction in rodents is associated with alterations in heart rhythm dynamics consistent with sympathoexcitation and parasympathetic withdrawal. Moreover, we report that mouse strain is an important variable when evaluating autonomic function via the analysis of HRV.

## Introduction

The autonomic nervous system modulates beating rate and modality of myocardial excitation, contraction, and relaxation allowing adjustments of cardiac performance to meet hemodynamic requirements under various conditions. Cardiac autonomic regulation occurs via feedback loop mechanisms involving, on the one hand, afferent sensory information transmitted from mechanoreceptors and chemoreceptors of heart and vasculature to intrathoracic ganglia and central nervous system. On the other hand, efferent cardiomotor neural impulses originating in the nervous system return to the heart via sympathetic and parasympathetic nerves, influencing heart rate, electrical conduction, and myocardial function (1, 2).

Sympathetic and parasympathetic stimulation, respectively, increases and decreases heart rate by modulating sinoatrial node discharge (3, 4). Norepinephrine, released by sympathetic nerves, binds β-adrenergic receptors activating adenylyl cyclase and cyclic AMP production, which induces faster diastolic depolarization and rapid firing rate. In contrast, acetylcholine, released by parasympathetic nerves, opens G-protein regulated K^+^ channels and binds muscarinic receptors that inhibit adenylyl cyclase, with the overall effect of reducing firing rate of the sinoatrial node. Although β-adrenergic and muscarinic receptors share common downstream transduction molecules, the pattern of beating rate adjustment following stimulation of the two signaling axes occurs with different kinetics. Specifically, parasympathetic activation has instantaneous and brief consequences on heart rate, whereas sympathetic stimulation has longer latency effects (5–9). Thus, based on the modalities of action of the two branches of the autonomic system on sinoatrial node discharge, the analysis of heart rate dynamics provides information on the influence of sympathetic and parasympathetic tone on the heart (5, 6, 9, 10).

Myocardial infarction and other cardiac pathologies interfering with pump function largely affect autonomic regulation. Acutely, sympathoexcitation and parasympathetic tone withdrawal allow for the preservation of cardiac output, but this initial response is often associated with chronic changes in the autonomic nervous system, involving abnormal cardiac afferent activity, excessive neuronal interactive excitability, and altered neuronal hierarchy (1, 2). The ensuing autonomic dysfunction together with neurohumoral activation, secondary to circulatory changes (11), are important factors in the progression of the diseased condition and enhanced vulnerability of the heart to arrhythmias (1, 12).

Heart rate variability (HRV) has been studied in patients suffering from myocardial infarction and indices of heart rate dynamics have been correlated to recurrent coronary events, cardiac arrhythmias, sudden cardiac death, and all-cause mortality (13, 14). Clinical investigations not only have strengthened the prognostic value of specific parameters of heart rate variability for patients with ischemic damage or other cardiovascular complications, but have also substantiated the prominent role of cardiac autonomic regulation in the initiation and progression of pathological conditions (2).

In the current study, we have attempted to clarify whether experimental animal models of cardiac disease recapitulate features of heart rate dynamics observed in humans, constituting valid platforms for the understanding of mechanisms linking heart rate variability and the development and manifestation of diseased conditions. Specifically, we have evaluated the acute and long terms consequences of myocardial infarction on heart rate dynamics in two mouse lines, using electrocardiograms (ECGs) collected in the conscious, restrained state, using a tunnel device (Emka Technologies). This analysis has been complemented with *ex vivo* studies in isolated organs to assess intrinsic beating rate of normal and infarcted hearts. Importantly, this investigation was conducted in mouse lines typically employed as control animals for studies allowing for conditional and inducible gene manipulations in the heart, as done previously by our group (15). These refined genetically engineered models represent remarkable tools for the elucidation of signaling pathways that underlie normal and diseased states, and have gained widespread use and recognition (16, 17). Our findings indicate that myocardial infarction reduces long- and short-term heart rate variability, which is consistent with enhanced sympathetic and reduced parasympathetic tone. Moreover, we found that, experimentally, mouse strain is an important variable when assessing heart rate variability in normal and pathological status.

## Materials and Methods

All data, materials, and methods of this study are available from the corresponding author upon reasonable request.

### Experimental Animals

Mice were maintained in accordance with the Guide for Care and Use of Laboratory Animals; animal experiments were approved by the local institutional animal care committees (IACUC) of New York Medical College. When needed, isoflurane (1–1.5%, inhalation) was employed as a methodology of anesthesia. Euthanasia was attained under anesthesia by bilateral thoracotomy and removal of the heart.

For this investigation, data collected from single transgenic mice homozygous or heterozygous for the alpha-MHC-MerCreMer (αMHC-MCM) transgene were used. In these animals, the mouse cardiac-specific alpha-myosin heavy chain promoter (αMHC) directs expression of a tamoxifen-inducible Cre recombinase (MerCreMer), allowing for the temporally regulated modulation of loxP-flanked targeted genes in cardiomyocytes of bi-transgenic mice (17). Mice homozygous or heterozygous for the αMHC-MCM transgene and presenting LoxP-flanked sequences have been widely employed for inducible and conditional gene targeting. Currently, according to the Web of Science database, there are >400 published research articles citing the original investigation reporting the development of the αMHC-MCM mouse line (17). Therefore, based on the relevance of this engineered mouse line, we employed homozygous and heterozygous αMHC-MCM animals, which are typically used as control mice in studies with conditional and temporal regulation of the expressions of genes of interest.

For this purpose, *B6.FVB (129)-A1cf^Tg^* ^(*Myh*6–*cre/Esr*1*)1*Jmk*^/*J* mice (αMHC-MCM, Jackson Labs, Stock No. 005657) (15, 17), backcrossed onto C57BL/6J inbred mice by the vendor to obtain a congenic strain, were utilized for breeding. Homozygous αMHC-MerCreMer mice (Homo) were obtained by inbreed crossing. In contrast, heterozygous αMHC-MerCreMer mice (Het) were generated by selecting the F1 hybrid progeny of mice originated from the crossing of αMHC-MCM homozygous animals with hemizygous ZEG-NICD mice (Jackson Labs, Stock No. 6850), originally derived on 129/Sv strain (15, 18). The F1 hybrid progeny consisted of single transgenic heterozygous αMHC-MCM mice and double transgenic heterozygous αMHC-MCM and ZEG-NICD mice born with normal Mendelian ratio of ∼1:1. From the progeny of the latter breeding scheme, only heterozygous αMHC-MCM mice and lacking ZEG-NICD transgene were used for this study. Therefore, no genes other than the αMHC-MCM were deleted or overexpressed in mice studied in this investigation. Moreover, C57Bl/6 mice and non-carrier mice used as control for the ZEG-NICD strain (wild-type) were introduced to provide information on the influence of genetic background on parameters studies in this investigation. C57Bl/6 mice were obtained from Charles River.

Cre recombinase expression in homozygous and heterozygous αMHC-MerCreMer mice was induced by administration of tamoxifen (Sigma-Aldrich) dissolved in 10% ethanol and 90% peanut oil (Sigma-Aldrich) for 4 days (30 mg/Kg of body weight/day, i.p.) over a period of 4–8 days (15, 19). The dose of tamoxifen was optimized to minimize off-target and confounding effects of drug administration and Cre recombinase expression in the heart, including the development of transient cardiomyopathy and DNA-damage response (19, 20). Mice were enrolled in the study at 3 or more weeks after induction of gene expression. Animals in the naïve state were studied before and after tamoxifen administration and Cre recombinase expression.

Unless otherwise specified, collected data were disaggregated by sex. Electrocardiographic analysis was performed in mice with age ranging from 2.4 to 6.6 months. For tests addressing the influence of the autonomic nervous system on heart rate dynamics of *ex vivo* perfused hearts, organs were obtained from homozygous αMHC-MerCreMer male and female mice at 4.3–8.5 months of age.

### Myocardial Infarction

Myocardial infarction was performed under sterile conditions via thoracotomy and coronary artery ligation (21, 22). Under isoflurane anesthesia (∼1.5%), the animal was intubated and ventilated continuously during the surgical procedure. A local anesthetic (lidocaine, ∼4 mg/kg) was injected subcutaneously in the incision site before and after the surgical procedure. The thorax was opened via the third intercostal space, the atrial appendage elevated. The left coronary artery was located, and suture (6–0 Silk DR12 Black 18” Braid, Henry Schein) was inserted around the vessel near the origin and the artery occluded. The chest was closed with suture and pneumothorax reduced by negative pressure. Skin incision was closed using a 9 mm wound clip (Fine Science Tool). To reduce postoperative pain following the procedure, buprenorphine hydrochloride (Buprenex), 0.5–1 mg/kg body weight, was injected i.p. every 12 h for a period of 48 h after surgery.

### Electrocardiographic Recording in the Conscious State

To record electrocardiograms (ECGs) in conscious animals an ECG-tunnel device (Emka Technologies) was employed (23–25). Animals were placed in a tunnel and ECGs recorded for a period of 10 min. Electrical signals were amplified with a 12 Lead ECG Amplifier (DSI, Ponemah), digitized using a 160 kHz A/D converter (DI-1120 HS, Dataq) and recorded with WinDaq software (Dataq). The bipolar lead I, II, and III and the unipolar lead aVL were collected. Electrical signals were evaluated offline with LabChart 8 for the analysis of heart rate variability and occurrence of rhythm disturbances (24).

### *In vivo* Cardiac Function

Echocardiography was performed in conscious mice, with singlehanded manual restraint method, using an Acuson Sequoia c512 equipped with a 13 MHz (15L8) linear transducer (26–29). By this approach, m-mode images in the parasternal short axis view of the left ventricle (LV) were employed to evaluate chamber diameter and wall thickness in diastole and systole, for computation of LV volume, mass, and ejection fraction (EF) by the Teichholz formula (26–29).

### Drugs

Effects of pharmacological compounds on heart rate and heart rate variability (HRV) were tested by comparing 10 min ECG recordings obtained before and after drug administration. In between acquisitions, mice were returned to their cages.

To interfere with autonomic nervous system, mice were administered with the combination of atropine (0.5 mg/kg body weight, i.p.) plus propranolol (1 mg/kg body weight, i.p.) for combined block of sympathetic and parasympathetic branches of the autonomic nervous system (combined autonomic block) (24, 28). Drugs were dissolved in USP saline solution. Effects of combined autonomic block were evaluated ∼10 min after drug(s) administration.

### *Ex vivo* Properties of the Mouse Heart

With the animal under deep anesthesia (isoflurane) and following administration of heparin (∼200 unit, i.p.), bilateral thoracotomy was performed, the ascending aorta was cannulated with PE50 tubing connected to a 23G 3/4 needle, and the heart was excised. Subsequently, hearts were perfused in a Langendorff apparatus (Radnoti). Perfusion was accomplished at a constant pressure of ∼80 mmHg with pre-warmed Krebs–Henseleit buffer (KHB; Sigma-Aldrich) containing, in mmol/L: 118 NaCl, 4.7 KCl, 11 glucose, 1.2 MgSO_4_, 1.2 KH_2_PO_4_, 1.8 CaCl_2_, and 25 NaHCO_3_, gassed with 95% O_2_ and 5% CO_2_ (pH 7.4) at 37^°^C (27–31). The temperature was maintained by immersing the heart in a water-heated glassware reservoir (Radnoti), containing preheated KHB.

To assess electrical activity of perfused hearts in sinus rhythm, two-lead mini ECG electrodes (Harvard Apparatus) were placed on the right atrium and apex of the left ventricle, respectively, to obtain pseudo-ECG (28–30) for 5–10 min. Electrical signals were amplified (6,600 Amplifier, Gould Instruments), digitized using a 160 kHz A/D converter (DI-1120 HS, Dataq) and recorded with WinDaq software (Dataq). Approximately 5 continuous minutes of ECGs collected in sinus rhythm were employed to assess heart rate dynamics of perfused hearts. At the completion of the procedure, the four chambers of the heart were dissected. A transverse section of the left ventricle was fixed in neutral buffered, 10% formalin solution (Sigma-Aldrich) for histological analysis. Electrophysiological data were analyzed offline with LabChart 8 software.

To assess the influence of intrinsic cardiac ganglia and neural terminations on heart rate dynamics of the excised, isolated heart, ECGs were obtained from organs perfused in the Langendorff system with KHB alone or in combination with 100 nM atropine or 1 μM propranolol. Concentration of these compounds was based on previous studies utilizing isolated or innervated (32) Langendorff perfused rodent hearts (33, 34).

### Settings for Data Analysis

Analysis of heart rate and heart rate variability (HRV) was conducted on electrocardiographic recordings using LabChart 8 (ADinstruments) and the HRV module, as reported previously (24). Lead I was used to obtain HRV parameters and other leads were employed as alternative source of analysis and/or for validation of obtained results. The entire 10 min of recording was analyzed. Company presets of LabChart HRV module for the mouse were adopted, with minor modifications of parameters for beat classification to include valid RR intervals detected by the QRS complex. Ectopic beats were excluded from the computation. A report, providing parameters of HRV, was generated by the software and values exported to Microsoft Excel for quantification.

### Parameters Employed to Describe Heart Rate Dynamics

The RR interval duration was quantified by the average of RR intervals (average RR) obtained during the 10 min of acquisition. Heart rate variability (HRV) was quantified by using time-domain and frequency domain variables, together with non-linear parameters obtained from the Poincaré plots (5, 6, 24, 35, 36). Specifically, time-domain variables included standard deviation of RR intervals (SDRR), coefficient of variation of RR intervals (CVRR, obtained by dividing SDRR by the average RR interval), square root of the mean of the squared differences between adjacent RR intervals (RMSSD, an index of short-term variability). Frequency-domain variables included total power (ms^2^), corresponding to energy in the entire power spectrum analyzed (0–5 Hz). The spectrum of oscillations of RR interval duration was separated in very low-frequency power (VLF, between 0 and 0.15 Hz), low-frequency power (LF, between 0.15 and 1.5 Hz), and high-frequency power (HF, between 1.5 and 5 Hz). Data for VLF, LF, and HF were computed as percentage of the total power (%). Moreover, low-frequency/high-frequency ratio (LF/HF, ms^2^/ms^2^) was computed. High-frequency components of RR interval variations are attributed to respiratory-mediated modulation of heart rate by the parasympathetic system, whereas low-frequency components are attributed to baroreflex-mediated modulation of heart rate (5, 24, 37). Very low-frequency components of RR interval variation are attributed to the modulatory action of the renin-angiotensin system, thermoregulation, and partly, parasympathetic activity (24, 37). It is generally accepted that low-frequency components are influenced by both the sympathetic and parasympathetic system (5) and LF/HF ratio is an index of sympathovagal balance, estimating the ratio of sympathetic to vagus nerve tone (5, 6). However, caution has to be exercised when considering LF/HF ratio as indicator of sympathovagal balance based on the fact that low-frequency components of RR interval variability are affected by sympathetic, parasympathetic, and other unidentified factors (10, 24). Non-linear parameters were obtained from the Poincaré plot, a graphical representation of RR interval (RR_*n*_) plotted against the next one (RR_n+1_) (38, 39). Standard deviation of instantaneous beat-to-beat interval variability (SD1) and standard deviation of continuous long-term RR interval variability (SD2) were obtained by an ellipse-fitting technique of plotted data. The SD1 index correlates with the short-term variability of RR interval variation and is mainly influenced by parasympathetic modulation whereas SD2 is a measure of long-term variability and reflects sympathetic activation (24, 39). The SD1/SD2 ratio is an index of autonomic balance and it inversely correlates with the LF/HF ratio (37).

### Histological Analysis and Assessment of Myocardial Infarct Size

Macroscopic images of formalin-fixed transverse sections of the left ventricle (LV) at the mid-ventricular level were obtained with a stereo microscope (SW-3T13X, Amscope) equipped with a digital camera (MU1003, Amscope). Images were employed to evaluate infarct size using the midline length approach (40). Briefly, using ImageJ software and collected images, a line was manually drawn at the center of the surviving myocardium between the epicardial and endocardial surfaces and at the level of the thin transmural scar tissue. The two lines related to surviving myocardium and scar tissue formed a circumference. Infarct size expressed in percentage was derived by dividing the length of midline at the level of the scar tissue by the total length of the circumference, and multiplying by 100.

For a subgroup of hearts, transverse sections of the LV at the mid-ventricular level were embedded in paraffin and sliced to obtain thin sections (∼4 mm thickness) (21, 28). For detection of fibrotic tissue, slides were trichrome-stained (Masson’s Trichrome Stain Kit, Mastertech, StatLab) following manufacturer’s instruction (28, 41). Images were acquired using a stereo microscope (SW-3T13X, Amscope) equipped with a digital color camera (MU1003, Amscope). Images were employed to evaluate scar size using the area approach (40). Briefly, using collected images, areas of the fibrotic tissue and total area of LV section were traced manually using ImageJ software. Fibrotic tissue size was expressed as percentage of the sum of fibrotic areas by the total LV area or the section.

### Statistical Analysis

Data are presented in the text as fold- or percentage- change based on ratio of median values. Graphically, data are presented as scattered plots with indication of median and interquartile ranges, unless otherwise specified. Statistical analysis was performed using GraphPad Prism 9. Data were initially tested for normality (Shapiro-Wilk) and equal variance for assignment to parametric or non-parametric analysis. Parametric tests included Student’s *t*-test or analysis of variance followed by Bonferroni test for non-paired comparison between two or among multiple groups, respectively. For paired statistical analysis, paired *t*-test was employed. When normality or equal variance were not met, analysis was performed using Mann–Whitney Rank sum test or Kruskal–Wallis one-way analysis of variance on ranks followed by Dunn’s method, for non-paired comparison between two or among multiple groups, respectively. Wilcoxon signed rank test was employed for paired comparisons (15, 24, 27–29, 31, 41). Fisher’s exact test and survival curve comparison with Gehan-Breslow-Wilcoxon and Log-rank (Mantel-Cox) tests were performed using GraphPad Prism software. *P* < 0.05 was considered significant. Graphs were prepared using GraphPad Prism.

## Results

### Heart Rate Dynamics in Naïve Mice

Heart rate variability was evaluated in naïve mice obtained from a transgenic line routinely employed in studies for temporal regulation of the expression of genes of interest in the heart. Specifically, single transgenic mice homozygous or heterozygous for a genetic construct allowing for expression of a tamoxifen-inducible Cre recombinase (MerCreMer, MCM) under the alpha-myosin heavy chain promoter (αMHC) were employed (αMHC-MCM) (15, 17). Animals were single transgenic and negative for loxP-flanked sequences. Thus, no genes other than Cre recombinase had purposely altered expression.

Homozygous αMHC-MCM^+/+^ mice (Homo) were derived by inbreed crossing of animals developed and maintained in a C57Bl/6 background. In contrast, heterozygous αMHC-MCM^±^ mice (Het) were selected from the single transgenic F1 hybrid progeny of Homo mice crossed with hemizygous ZEG-NICD animals, derived on the 129/Sv strain (15, 18). Double heterozygous mice derived from the latter breeding scheme were not included in the study. Thus Homo and Het mice employed in this investigation differed for the number of MerCreMer alleles and for the hybrid genetic background introduced with the ZEG-NICD strain in Het hybrid mice.

**FIGURE 1:**
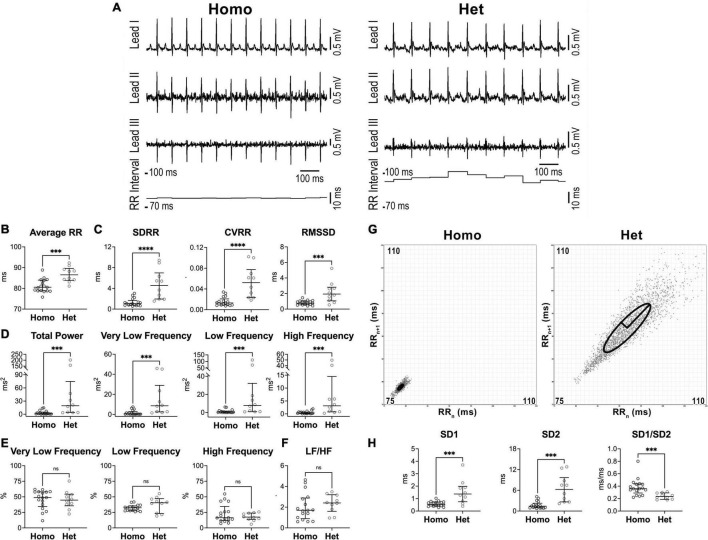
Heart rate dynamics in naïve female Homo and Het mice. **(A)** ECGs recorded in Homo and Het female mice. Traces for the three leads and RR interval duration are reported. **(B)** Quantitative data for average RR interval duration. **(C)** Data for time-domain parameters of HRV. SDRR, standard deviation of RR intervals; CVRR, coefficient of variation of RR intervals; RMSSD, square root of the mean of the squared differences between adjacent RR. **(D)** Data for frequency-domain parameters of HRV. **(E)** Data for frequency bands normalized by total power. **(F)** Data for LF/HF ratio. **(G)** Poincaré plots obtained from ECGs recorded from female Homo and Het mice reported in A. RR_*n*_ and RR_*n*+1_ axes span from 75 to 110 ms. **(H)** Data for non-linear indices. Quantitative data were obtained from naïve Homo (*n* = 17) and Het (*n* = 10) female mice. Data are shown as scattered plots with median and interquartile ranges. ns, not significant, ^***^P < 0.001, ^****^P < 0.0001 using unpaired *t*-test or Mann-Whitney test.

Parameters of heart rate variability were obtained from electrocardiographic recordings in conscious, restrained Homo and Het mice maintained in a tunnel device over a period of 10 min (25). With respect to sex-matched Homo animals, female Het mice presented prolonged average RR interval duration (+7%) (Figures 1A,B) and enhanced RR interval variation, quantified by standard deviation of RR intervals (SDRR, 4.3-fold increase), the coefficient of variance of RR intervals (CVRR, 4-fold increase), and the square root of the mean of the squared differences between adjacent RR intervals (RMSSD, 2.6-fold increase), which reflects short-term variability (Figure 1C).

By frequency-domain analysis, total power and very low-, low-, and high-frequency components of RR interval variations were larger in Het female mice with respect to sex-matched Homo animals (Figure 1D). However, the relative distribution of the three frequency components and the LF/HF ratio, which is generally accepted as an index of sympathovagal balance (5, 6), were similar in the two groups of mice (Figures 1E,F).

By Poincaré plots and non-linear analysis, beat-to-beat (SD1) and long-term (SD2) RR interval variability were, respectively, 2.6- and 4.4-fold larger in Het female mice with respect to sex-matched Homo animals, whereas SD1/SD2 ratio, generally accepted an index of autonomic balance (37), was reduced (Figures 1G,H).

Differences in parameters of RR interval duration and heart rate variability observed between Homo and Het female mice were also detected in cohorts of male animals (Supplementary Figure 1).

Because Homo and Het animals had different genetic background, a group of C57Bl/6 mice and wild-type (non-carrier, WT) mice derived on the 129/Sv strain (18) were studied. With respect to C57Bl/6, female WT mice had prolonged RR interval duration and enhanced RR interval variation, as quantified by SDRR (7.2-fold increase), CVRR (5.3-fold increase), and RMSSD (13-fold increase) (Supplementary Figures 2A,B). Similarly, total power and very low-, low-, and high-frequency components of RR interval variations were larger in female WT mice, with respect to sex-matched C57Bl/6 animals (Supplementary Figure 2C). However, the relative distribution of the three frequency components and the LF/HF ratio, were comparable between the animals with different genetic background (Supplementary Figures 2D,E). By Poincaré plots and non-linear analysis, SD1 and SD2 were, respectively, 13- and 6.7-fold larger in female WT mice with respect to sex-matched C57Bl/6 animals (Supplementary Figure 2F). Similar results were obtained in male mice (Supplementary Figure 3).

Subsequently, to evaluate the influence of genetic background on heart rate dynamics of Homo and Het mice, parameters of heart rate variability for C57Bl/6, Homo (C57Bl/6 background), Het (hybrid C57Bl/6 and 129/Sv background) and WT (129/Sv background) mice were compared. For this analysis data for male and female animals were combined. Overall, for time-domain, frequency domain, and non-linear parameters, C57Bl/6 and Homo mice behaved similarly, but differed from Het and WT animals. In contrast, Het and WT animals had a comparable behavior in the settings of the multiple comparison test (Supplementary Figure 4). Similar results were obtained when data was disaggregated by sex (data not shown).

Therefore, inbred homozygous mice for the MerCreMer transgene present reduced RR interval duration and attenuated short- and long-term heart rate variability with respect to hybrid heterozygous animals. Similar trends were observed between C57Bl/6 and WT mice, suggesting that mouse genetic background represents a variable when considering heart rate dynamics.

### Autonomic Regulation and Heart Rate Dynamics in Naïve Mice

**FIGURE 2:**
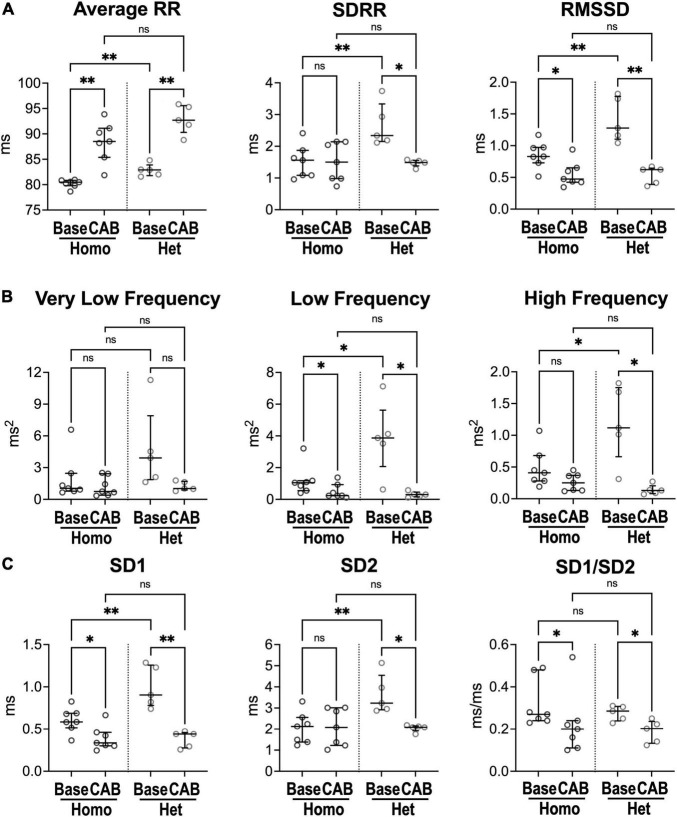
Inhibition of the autonomic nervous system and heart rate dynamics in naïve Homo and Het mice. **(A)** Quantitative data for RR interval duration and time-domain parameters of HRV for Homo and Het mice before (baseline, Base) and after combined autonomic block with atropine and propranolol (CAB). **(B)** Data for frequency-domain parameters of HRV. **(C)** Data for non-linear indices. Quantitative data were obtained from naïve Homo (*n* = 7) and Het (*n* = 5) female mice. Data are shown as scattered plots with median and interquartile ranges. ns, not significant, *P < 0.05, ^**^P < 0.01 using paired *t*-test or Wilcoxon signed rank test for comparisons within each genotype and unpaired *t*-test or Mann-Whitney test for comparisons across genotypes.

To establish the contribution of autonomic nervous tone and circulating catecholamines to differences of heart rate dynamics in homozygous and heterozygous animals studied here, heart rate variability (HRV) was evaluated in the presence of blockers of muscarinic and beta-adrenergic receptors, to inhibit the effects of sympathetic/parasympathetic branches of the autonomic nervous system and circulating factors. Specifically, electrocardiographic recordings were obtained in naïve mice before (baseline, Base) and after combined autonomic block (CAB), which was achieved by administration of atropine, a muscarinic receptor antagonist, and propranolol, a beta-adrenergic receptor blocker (24, 28). CAB prolonged average RR interval duration (+10%, +12%) and reduced RMSSD (−43%, −51%) in both Homo and Het female mice, but attenuated SDRR only in Het mice (−36%) (Figure 2A). Additionally, in Het mice, CAB reduced low- (−92%) and high-frequency (−89%) oscillations of RR interval duration (Figure 2B), whereas only low-frequency components were reduced in Homo mice (−77%). For non-linear parameters, CAB affected SD1/SD2 ratio by reducing SD1 in both groups of mice, but SD2 only in Het mice (Figure 2C). Importantly, CAB abrogated differences of RR interval duration, time-domain parameters, and non-linear SD1 and SD2 observed between Homo and Het mice with intact autonomic nervous function. Moreover, CAB was equally effective in abrogating differences in heart rate variability observed between C57Bl/6 and WT mice (Supplementary Figure 5).

Thus, inhibition of sympathovagal tone response renders HRV parameters comparable in homozygous and heterozygous mice, as well as in C57Bl/6 and WT animals, suggesting that differences in heart rate dynamics in mice with different genetic background are secondary, at least in part, to altered autonomic nervous inputs.

### Sex and Cre Recombinase Expression and Heart Rate Dynamics in Naïve Mice

Studies in humans and experimental models have documented that sex is an important biological variable in the physiology of the heart, in normal and diseased conditions (42–45). Thus, to define whether sex affects heart rate dynamics in the cohort of Homo and Het mice, RR interval parameters obtained from male and female naïve animals were compared. No significant differences were observed between the two sexes for each genotype, with respect to RR interval duration, time- and frequency-domain parameters, and non-linear variables (Supplementary Figures 6, 7).

Moreover, based on the genetic construct of the experimental model employed here, heart rate dynamics in Homo and Het mice was compared in cohorts of naïve mice non-treated or treated with tamoxifen, to induce Cre recombinase expression. When considering male and female animals together, differences observed between Homo and Het non-treated mice for RR interval duration and parameters of HRV were maintained following tamoxifen (Tmxf) treatment (Supplementary Figure 8). Additionally, following tamoxifen administration, male and female mice for each genotype had comparable parameters of HRV (data not shown).

To establish whether differences in heart rate dynamics observed in Homo and Het mice were coupled with alterations of cardiac performance, echocardiography was conducted. Sex- and age-matched Homo and Het mice had comparable left ventricular (LV) end-diastolic volume, ejection fraction, and cardiac output (Supplementary Figures 9A,B). LV mass was larger in male Het mice with respect to their female counterparts and with respect to male Homo animals. However, normalization of LV mass by chamber volume or body weight abrogated these differences (Supplementary Figure 9C).

Therefore, for each genotype, heart rate dynamics are comparable between male and female mice. Moreover, Homo and Het mice maintain different behavior of HRV in the absence and presence of Cre recombinase expression.

### Myocardial Infarction and Heart Rate Dynamics in Mice

To establish the consequences of myocardial infarction (MI) on heart rate and its variability, permanent coronary artery ligation (21) was performed in Homo and Het mice. Electrocardiograms were then collected at regular time intervals up to ∼1 month after induction of the ischemic insult. Male and female mice were used in combination.

**FIGURE 3:**
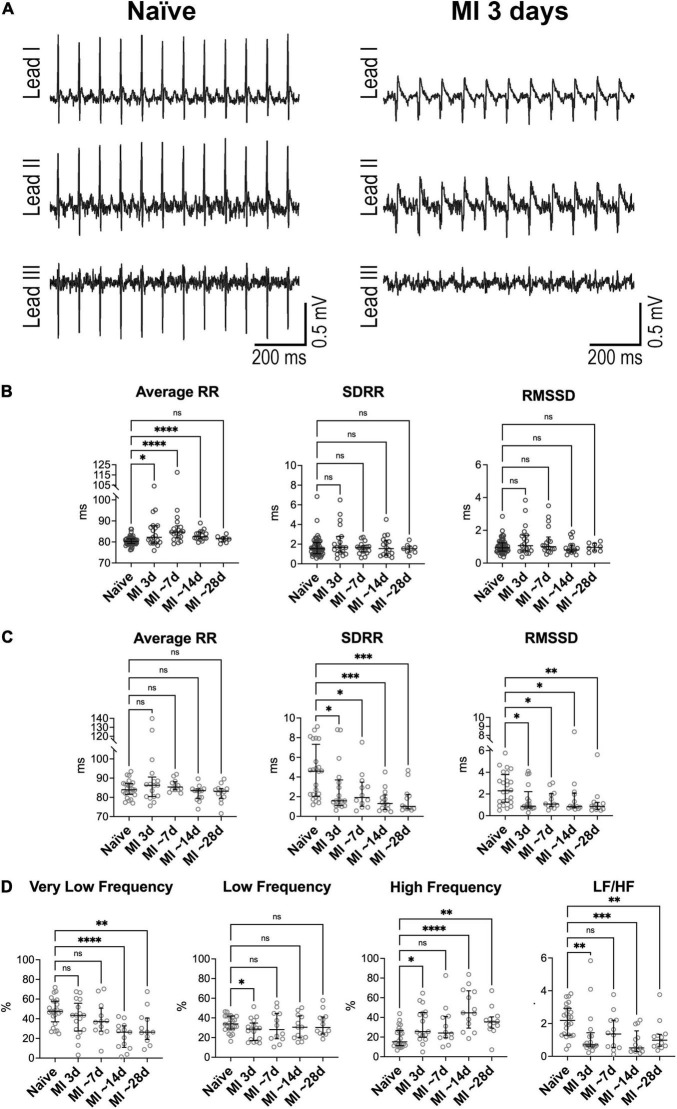
Heart rate dynamics in mice following myocardial infarction in Homo and Het mice. **(A)** ECGs obtained from a Homo mouse before (Naïve) and at 3 days after myocardial infarction (MI). **(B)** Quantitative data for average RR interval duration and time-domain parameters of HRV in Homo naïve male and female mice (*n* = 53) and Homo male and female mice at 3 (*n* = 19), 7–9 (∼7, *n* = 18), 13–15 (∼14, *n* = 16), and 28 (∼28, *n* = 8) days after MI. **(C,D)** Quantitative data for average RR interval duration, time-domain parameters **(C)** and frequency-domain indices **(D)** of HRV in Het naïve male and female mice (*n* = 24) and Het male and female mice at 3 (*n* = 17), 7–8 (∼7, *n* = 12), 13–15 (∼14, *n* = 13), and 29–33 (∼28, *n* = 12) days after MI. Data are shown as scattered plots with median and interquartile ranges. ns, not significant, *P < 0.05, ^**^P < 0.01, ^***^P < 0.001, ^****^P < 0.0001 using unpaired *t*-test or Mann-Whitney test.

With respect to the naïve state, ECG waveforms were substantially affected in mice following myocardial infarction (Figure 3A). Specifically, QRS complex, for the various collected leads, presented abnormal orientation in infarcted animals, a feature consistent with lack of electrical activation of the damaged myocardium. By echocardiography at 2 and 4 weeks after coronary artery ligation, all infarcted animals presented akinetic anterior LV free wall (Supplementary Figure 10A), low ejection fraction, and dilated LV chamber. Importantly, Homo and Het infarcted animals had comparable LV ejection fraction, cardiac output, and LV mass. However, with respect to Homo mice, LV end-diastolic volume was increased, and the posterior wall thickness-to-chamber radius ratio was reduced in Het mice (Supplementary Figure 10B). Infarct size, evaluated by measuring the fraction of the thin, scarred tissue with respect to the entire myocardial transverse sections of the LV at the mid-ventricular level, was ∼39% in Homo and ∼44% in Het mice (Supplementary Figure 11A). For a subgroup of animals, sections were processed histologically and trichrome-stained for the identification of fibrotic tissue (Supplementary Figure 11B). The fibrotic tissue constituted ∼33 and 28% of the total area of the LV section for Homo and Het hearts (Supplementary Figure 11C).

By electrocardiographic analysis, low heart rate was transiently observed at ∼24 h after the surgical procedure, when animals were under buprenorphine treatment. Specifically, at 1 day after MI, RR interval duration increased by 16% in Homo and 19% in Het mice, with respect to naïve animals of the same genotype. This behavior, which is consistent with the reported drop in heart rate secondary to buprenorphine treatment in mice at postoperative day 1 (46), precluded the consideration of this time point for evaluation of heart rate variability.

In Homo mice, with respect to naïve animals of the same genotype, RR interval duration was prolonged at 3 days (+2%), ∼7 days (+6%), and ∼14 days after MI (+3%). In contrast, long- and short-term RR interval variation, quantified by SDRR and RMSSD, respectively, were not affected at after MI (Figure 3B). The relative contribution of low-frequency components of RR interval variation was reduced at 3 days after MI, whereas at ∼7 days, contribution of very low-frequency was attenuated and high-frequency was increased (Supplementary Figure 12). No changes were observed for non-linear parameters. When data for Homo infarcted mice were disaggregated by sex, overall comparable results were seen (data not shown).

In Het mice, with respect to naïve animals of the same genotype, RR interval duration was not altered from 3 to ∼28 days after MI. In contrast, both SDRR and RMSSD were decreased with respect to naïve mice by ∼58–78% at time points comprised between 3 and ∼28 days after MI (Figure 3C). Additionally, the relative contribution of very low-frequency components of RR interval variation was reduced at ∼14 and ∼28 days after MI with respect to naïve animals, whereas high-frequency components were increased, with consequent attenuation of LF/HF ratio (Figure 3D). The non-linear parameter SD1 and SD2 followed the pattern of reduction observed for time-domain indices (Supplementary Figure 13). When data for Het infarcted mice were disaggregated by sex, overall similar results were detected (data not shown).

Differences in RR interval duration, short- and long-term RR variability, and total power of RR interval variation between Homo and Het mice observed in the naïve state (see Figure 1 and Supplementary Figures 1, 8) were abrogated at 3–∼28 days after induction of MI. In contrast, the relative contribution of high-frequency band of RR interval variation and of LF/HF ratio, that were comparable between naïve Homo and naïve Het mice, became different for the two groups of mice at ∼14–28 days after MI (Supplementary Figure 14).

Therefore, myocardial infarction in Het, but not in Homo mice, attenuates long- and short-term RR interval variation, affects the relative contribution of very low- and high-frequency components of RR interval oscillations, and alters sympathovagal inputs in the settings of measurements in the conscious, restrained state.

### Myocardial Infarction and Intrinsic Heart Rate Dynamics of the Mouse Heart

To better define putative mechanisms underlying differences of heart rate dynamics observed *in vivo* in Homo and Het mice in the naïve state and with chronic myocardial infarction, hearts obtained from naïve animals and animals at ∼1 month after MI were studied using a Langendorff system. Male and female mice were used in combination.

**FIGURE 4:**
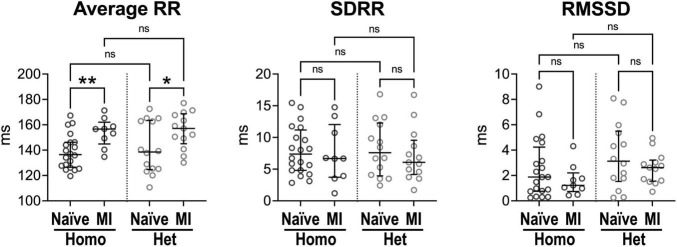
Intrinsic beating rate dynamics in normal and infarcted Homo and Het hearts. Quantitative data for average RR interval duration and time-domain parameters of HRV in naïve and infarcted hearts from Homo (*n* = 20, *n* = 9) and Het (*n* = 14, *n* = 13) male and female mice. Data are shown as scattered plots with median and interquartile ranges. ns, not significant, *P < 0.05, **P < 0.01, using unpaired *t*-test or Mann-Whitney test.

*Ex vivo*, isolated hearts from Homo and Het naïve animals had comparable intrinsic RR interval duration, SDRR and RMSSD (Figure 4). For both Homo and Het groups, intrinsic RR interval duration was increased in infarcted hearts (+15 and +13%, respectively) in comparison to corresponding non-infarcted organs. However, RR interval variation, assessed by SDRR and RMSSD, was not affected.

In the attempt to establish the contribution of intrinsic cardiac ganglia (33, 47) on RR interval properties of explanted, perfused hearts, organs were studied in the absence or presence of muscarinic or beta-adrenergic receptor inhibitors. Duration of RR interval, SDRR, and RMSSD were comparable for heart perfused without receptor blockers, with atropine, or with propranolol (Supplementary Figure 15). These results suggest that intrinsic cardiac ganglia have limited influence on the firing rate of the sinoatrial node, under experimental conditions employed here.

Therefore, in the absence of higher centers of autonomic nervous system modulation and extrinsic factors, intrinsic heart rate dynamics is comparable in Homo and Het intact organs. Chronic infarct increases RR interval duration in hearts from Homo and Het mice, suggesting that the diseased condition affects mechanisms of pacemaker discharge.

### Myocardial Infarction and Occurrence of Adverse Events

To establish whether the peculiar behavior of heart rate dynamics in Homo and Het mice was associated with the propensity of animals to develop adverse events following MI, occurrence of premature ventricular complexes (PVCs) and ventricular tachycardia (VT) were evaluated from electrocardiographic recordings. Moreover, post-MI survival was computed for the two groups of animals. Male and female mice were used in combination.

**FIGURE 5:**
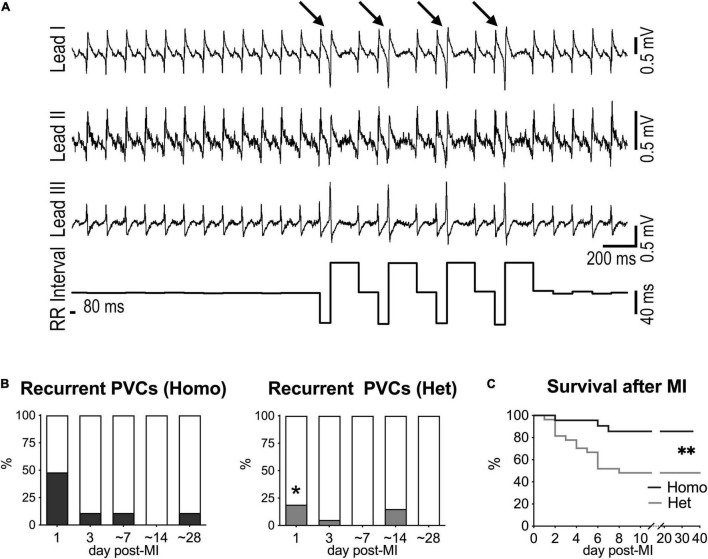
Myocardial infarction and occurrence of ectopic events in Homo and Het mice. **(A)** ECGs recorded in a Homo female mouse at 1 day after MI. Traces for the three leads and RR interval duration are reported. Arrows indicate PVCs. **(B)** Quantitative data for occurrence of recurrent PVCs (> 10 PVCs/10 min) at 1, 3, ∼7, ∼14, and ∼28 days after MI in Homo (*n* = 23, 19, 18, 16, 9, respectively) and Het (*n* = 27, 20, 13, 13, 13, respectively) male and female mice. *P < 0.05 vs. Homo, using Fisher’s exact test. **(C)** Survival curves for Homo (*n* = 23; 14 males, 9 females) and Het (*n* = 27; 10 males, 17 females) mice following MI. ^**^P < 0.01, using Gehan–Breslow–Wilcoxon and log-rank (Mantel–Cox) tests. In the cohort of Het mice, survival for male and female animals following MI was 40 and 53%, respectively.

Ventricular ectopic events were not detected in naïve mice (data not shown), but isolated or recurrent PVCs (>10 PVCs/10 min) together with VT were observed following myocardial infarction. Specifically, at 1 day after MI, recurrent PVCs were observed in 48% of Homo and 19% of Het mice, but occurrence of ectopic event progressively disappeared at later time points (Figures 5A,B). Also, VT was only detected at 1 day after MI in 17% of Homo and 11% of Het mice. For both Homo and Het mice at 1 day after MI, HRV parameters were overall comparable in mice without or with recurrent PVCs (Supplementary Figure 16).

Importantly, survival after MI was significantly reduced in Het mice with respect to Homo animals (Figure 5C). Parameter of HRV evaluated at 1 and 3 days after MI for each genotype did not discriminate between animals that survived or did not-survive (data not shown).

Thus, Homo and het mice have a different propensity to develop ectopic events and to survive in the early phase following myocardial infarction.

## Discussion

Results of the current study document that myocardial infarction in rodents affects heart rate dynamics and influences intrinsic sinoatrial node discharge. Interestingly, the use of two mouse lines has allowed us to identify, under our experimental conditions, strain-related differences of heart rate variability in the naïve state, which appear to be secondary, at least in part, to altered tone of sympathetic and parasympathetic inputs. These dissimilarities in autonomic function partly hinder the manifestation of autonomic imbalance induced by myocardial damage.

Studies conducted in male and female naive animals revealed that, for mouse lines studied here, sex has minor effects on heart rate dynamics, indicating that sympathovagal balance in male and female mice is comparable when evaluated in the conscious, restrained state, using the tunnel device. Moreover, RR interval duration and heart rate variability were comparable between mice homozygous for the αMHC-MerCreMer transgene (Homo) and syngeneic C57Bl/6 mice, suggesting that the transgene does not interfere with heart rate dynamics. Additionally, we found that mice with homozygous or heterozygous expression of Cre recombinase have heart rate variability comparable to animals of the same genotype before induction of gene expression. Overall, these findings suggest that the αMHC-MerCreMer transgene and Cre recombinase expression, in the homozygous and heterozygous state, do not affect heart rate dynamics.

Inbred Homo mice, used in this study, were developed and maintained in a C57Bl/6 background (15, 17), whereas Het hybrid mice were obtained by crossing Homo animals with mice originally derived in the 129/Sv strain (18). We found that, with respect to Homo animals, both male and female Het naïve mice have increased RR interval duration and enhanced heart rate variability, as assessed by time-domain, frequency-domain, and non-linear parameter of RR interval variation. Importantly, these differences were abrogated following combined autonomic block, strengthening the role of sympathovagal inputs in modulating heart rate dynamics in mouse of the two lines. Moreover, *ex vivo* tests revealed that RR interval duration and time-domain parameters of variability are comparable in Homo and Het hearts deprived from the influence of higher centers of the autonomic nervous system. Thus, these findings suggest that the two groups of mice have different autonomic behavior under experimental conditions employed here. Specifically, Het mice, with respect to Homo, appear to have lower sympathetic and/or increased parasympathetic input. Importantly, differences for heart rate variability between C57Bl/6 mice and non-carrier, wild-type mice, derived on the 129/Sv strain, are consistent with alterations observed between Homo and Het mice. These results point to mouse strain as an important variable in modulating heart rate dynamics.

It is recognized that differences exist between C57Bl/6 and 129/Sv strains in relation to behavioral traits and autonomic response to stress and anxiety (48, 49). It has been reported that 129/Sv have attenuated increase in heart rate in response to a mild-intensity stressor, with respect to C57Bl/6 mice (49). These findings are consistent with observed differences in RR interval duration and RR interval variability between naïve C57Bl/6 and WT mice, as well as Homo and Het mice, under forced immobilization during ECG collection, a condition that induces physical and emotional stress (24, 50, 51). Thus, differences of HRV observed between Homo and Het mice appear to be secondary to autonomic modulation pertaining to the strain of these animals, a factor that may be relevant when assessing, experimentally, sympathovagal tone in response to stressors or pathological conditions.

Clinical studies have documented that various cardiac pathologies, including myocardial infarction, are coupled with perturbations of autonomic regulation that manifest with reduced HRV (2, 13). It has been reported that, in patients with MI, reduced short-term heart rate variability and declined very low- and low-frequency spectral components of RR interval variation correlate with poor prognosis and increased risk of all-cause mortality (13, 52–54). Moreover, very low-frequency components are associated with fatal or near fatal arrhythmic events in individuals with MI and depressed left ventricular function (55). Interestingly, with respect to patients at 2 weeks after acute myocardial infarction, spectral components of HRV were found to be partly restored in individuals at 6 and 12 months after the acute event, suggesting a progressive recovery of vagal tone and a normalization of sympathovagal interaction (56). Thus, in patients with myocardial infarction, the analysis of heart rate dynamics predicts future outcomes and provides information on the level of progression/recovery from the diseased state.

Experiments performed here have allowed us to establish alterations of heart rhythm dynamics occurring in mice following myocardial infarction, and to evaluate the validity of this rodent model in recapitulating features of autonomic regulation in humans with ischemic disease. In Homo mice, characterized by high beating rate and low RR interval variability in the naïve state, which is consistent with high sympathetic input, no major changes in HRV parameters were induced after MI, except for the prolongation of RR interval duration observed at 3–∼14 days after coronary ligation. Prolonged RR intervals were also found in explanted infarcted hearts, with respect to non-infarcted organ. Together, these data suggest that the high level of sympathetic activation observed in homo naïve animals may have blunted sympathoexcitation secondary to the ischemic damage, preventing increases in heart rate and revealing the intrinsic prolongation of RR interval duration of the infarcted heart. In contrast, in Het mice, myocardial infarction was associated with reduced long and short-term RR interval variation, consistent with sympathoexcitation and parasympathetic tone withdrawal. The lack of changes in RR interval duration following MI in Het mice may have resulted from the combination of sympathetic activation and reduced parasympathetic input counteracting the prolongation of the intrinsic RR interval, which was observed in isolated infarcted organs. Importantly, Het mice following MI had reduced short-term RR variability and attenuation of very low-frequency and, in part, low-frequency oscillations, reiterating alterations observed in human with ischemic disease.

Interestingly, HRV parameters were comparable in Homo and Het mice following myocardial infarction, suggesting that common autonomic adaptations occurred in the two mouse lines in response to the ischemic damage. However, at ∼14 and ∼28 days after MI, Het mice had increased relative contribution of high-frequency components of RR interval variation and decreased LF/HF ratio, with respect to Homo infarcted animals. These differences may partly reflect adjustments of autonomic control in response to the evolution of the diseased condition for the two mouse lines. In this regard, although cardiac function was equally depressed in the two groups at 2 and 4 weeks after MI, Het mice had higher degree of LV dilation and reduced wall thickness/chamber radius ratio with respect to Homo animals, which are consistent with maladaptive remodeling.

Time-domain, frequency-domain, and non-linear analyses provide complementary and corroborating information on properties of heart rate and putative regulatory mechanisms active under various conditions. In Het mice, following myocardial infarction, both long- and short-term RR interval variation, quantified by SDRR and RMSSD, were reduced. Non-linear analysis led to similar findings, as indicated by the reduced SD1 and SD2. While both sympathetic and parasympathetic inputs affect long-term variability, short-term RR interval variation is mainly under vagal influence (37), suggesting that alterations of the two inputs of the autonomic nervous system occurred after MI. Frequency-domain analysis not only provided indication that heart rate properties were altered after infarction, but also revealed that the relative contribution of very low-frequency bands (RR interval variations occurring at frequencies <0.15 Hz) were reduced whereas contribution of high-frequency bands (RR interval variations occurring at frequencies 1.5–5 Hz) increased. As previously discussed (see “Materials and Methods” section), very low-frequency components of RR interval variation are attributed to the modulatory action of the renin-angiotensin system, thermoregulation, and, partly, parasympathetic activity, whereas high-frequency components of RR interval variations are attributed to respiratory-mediated modulation of heart rate by the parasympathetic system (5, 24, 37). Overall, the time course analysis of heart rate variability and combination of time- and frequency-domain parameters appear to reflect the complex adaptations associated with the acute and chronic phases of myocardial infarction (57).

Studies addressing the effects of myocardial infarction on HRV in rodents are limited. Previous investigations using FVB (36) and C57Bl/6 (58) mice suggested that autonomic nervous system function, measured by indices of HRV from ECGs recorded by telemetry, was not altered during ischemia or infarction. Interestingly, conscious heart rate is higher in FVB and C57Bl/6 mice with respect to 129/Sv animals (59), and this factor favors the possibility that enhanced sympathetic input in FVB and C57Bl/6 mice may have masked sympathoexcitation following the ischemic damage, as document for the Homo mouse line studied here. Similarly, no changes were detected in HRV parameters in Sprague-Dawley rats at 28 and 56 days after coronary artery ligation, with respect to non-infarcted animals (60). However, at 3 and 28 days after MI, baroreflex sensitivity was found to be transiently reduced, as determined by decreased reflex bradycardia (60). Moreover, in a separate study using Wistar rats, standard deviation of beat-to-beat interval duration, low-frequency components, and LF/HF ratio were found to be reduced at ∼3 months after MI (61). Thus, while clinical studies document an association between myocardial infarction and reduced HRV (2, 13), this relationship appears to be less apparent in small experimental animals. Our results tend to suggest that sympathetic predominance and low vagal tone in naïve rodents may represent key factors hindering the manifestations of sympathoexcitation and sympathetic withdrawal on HRV, in the settings of MI.

Homo and Het infarcted mice used in our investigation presented different propensity to develop ectopic ventricular events, mainly detected at 1 day after the surgical procedure. This time point, however, coincided with the treatment of animals with analgesia, which exerts negative chronotropic effects on the heart (46) and imposes caution on data interpretation. Although animals with ectopic events tended to have longer RR interval duration with respect to mice without PVCs, no major differences were observed for parameters of HRV. Thus, as previously reported for rodent models of aging (24), for mice with myocardial ischemic damage there is no clear correlation between heart rate variability and occurrence of ectopic ventricular events.

Post-MI survival was found to be higher in Homo mice with respect to Het, a behavior that appears to be consistent with reported strain-related differences in ventricular rupture, which in mice occurs within the first week after myocardial infarction (45, 62). Specifically, previous studies documented that occurrence of myocardial laceration is higher in 129/Sv mice with respect to C57Bl/6 animals (45, 62). Although we have not evaluated whether acute heart failure or myocardial rupture affected survival of animals studied here, our findings align with the notion that our Het hybrid mice, derived from crossing 129/Sv background, are more vulnerable to myocardial laceration and subsequent death.

In the current investigation, we did not directly address factors possibly involved in the alterations of heart rate dynamics observed in mice following myocardial infarction. In this regard, remodeling of intrathoracic ganglia and/or intrinsic cardiac nervous system, including ganglia located in the right atrium (63–66), may have affected sympathetic and parasympathetic inputs at the level of the sinoatrial node of mice studied here. Moreover, in addition to variations of locally released neurotransmitters (67–69), circulating catecholamines, which are increased after myocardial infarction (70–72), may have contributed to the reduction of heart rate variability observed in Het mice following coronary artery ligation. Thus, the observed effects are likely multifactorial in origin.

## Conclusion

In conclusion, our study document that myocardial infarction in rodents is associated with alterations in heart rhythm dynamics consistent with sympathoexcitation and parasympathetic withdrawal. Moreover, we found that mouse strain is an important variable when evaluating autonomic function via HRV, a factor that may interfere with the assessment of the consequence of cardiac pathologies on autonomic regulation.

## Data Availability Statement

The raw data supporting the conclusions of this article will be made available by the authors, without undue reservation.

## Ethics Statement

The animal study was reviewed and approved by the New York Medical College IACUC.

## Author Contributions

MR conceived, designed the research, and drafted the manuscript. EP, SB, SJ, JJ, and MR designed and prepared the reagents. EP, SB, DC, VD, and MR performed the experiments and acquired the data. EP, SB, RK, DC, VD, and MR analyzed the data, performed statistical analysis, and interpreted results. SJ, JJ, and MR provided funding support and experimental resource. EP, SB, RK, DC, and MR edited and revised the manuscript. EP, SB, RK, DC, VD, SJ, JJ, and MR approved the manuscript. All authors contributed to the article and approved the submitted version.

## Conflict of Interest

The authors declare that the research was conducted in the absence of any commercial or financial relationships that could be construed as a potential conflict of interest.

## Publisher’s Note

All claims expressed in this article are solely those of the authors and do not necessarily represent those of their affiliated organizations, or those of the publisher, the editors and the reviewers. Any product that may be evaluated in this article, or claim that may be made by its manufacturer, is not guaranteed or endorsed by the publisher.
