# Supplementary material for: Heart Rate Variability Reveals Altered Autonomic Regulation in Response to Myocardial Infarction in Experimental Animals
Source: Front Cardiovasc Med. 2022 May 2;9:843144. doi: 10.3389/fcvm.2022.843144 (PMC9108187; doi:10.3389/fcvm.2022.843144)
Supplement: Supplementary file 1 [file Data_Sheet_1.pdf]

Heart Rate Variability Reveals Altered Autonomic Regulation in Response to Myocardial Infarction in Experimental Animals

Emanuele Pizzo,[1] Silvia Berrettoni,[1] Ridhima Kaul,[1] Daniel O. Cervantes,[1] Valeria Di Stefano,[1] Sudhir Jain,[2] Jason T. Jacobson,[1,3] and Marcello Rota[1]

[1]Department of Physiology, New York Medical College, Valhalla, NY, USA; [2] Department of Pathology, Microbiology and Immunology, New York Medical College, Valhalla, NY, USA; [3]Department of Cardiology, Westchester Medical Center, Valhalla, NY, USA

SUPPLEMENTAL FIGURES

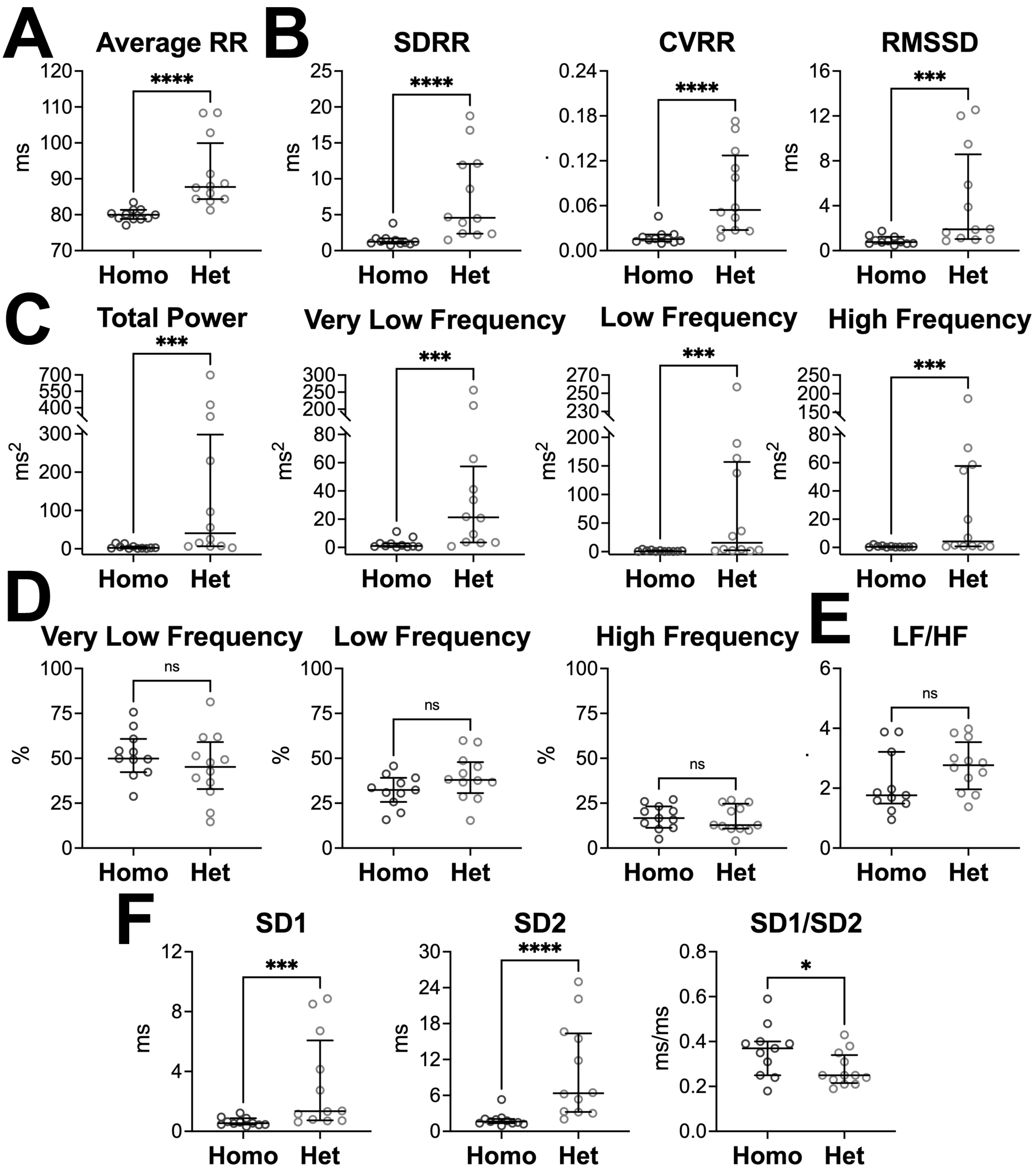

Supplementary Figure 1 | Heart rate dynamics in naïve male Homo and Het mice. (A) Quantitative data for average RR interval duration. **(B)** Data for time-domain parameters of HRV. SDRR, standard deviation of RR intervals; CVRR, coefficient of variation of RR intervals; RMSSD, square root of the mean of the squared differences between adjacent RR. **(C)** Data for frequency-domain parameters of HRV. **(D)** Data for frequency bands normalized by total power. **(E)** Data for LF/HF ratio. **(F)** Data for nonlinear indices. Quantitative data were obtained from naïve Homo (n = 11) and Het (n = 12) male mice. Data are shown as scattered plots with median and interquartile ranges. ns, not significant, $^{*}P < 0.05$, $^{***}P < 0.001$, $^{****}P < 0.0001$ using unpaired *t*-test or Mann-Whitney test.

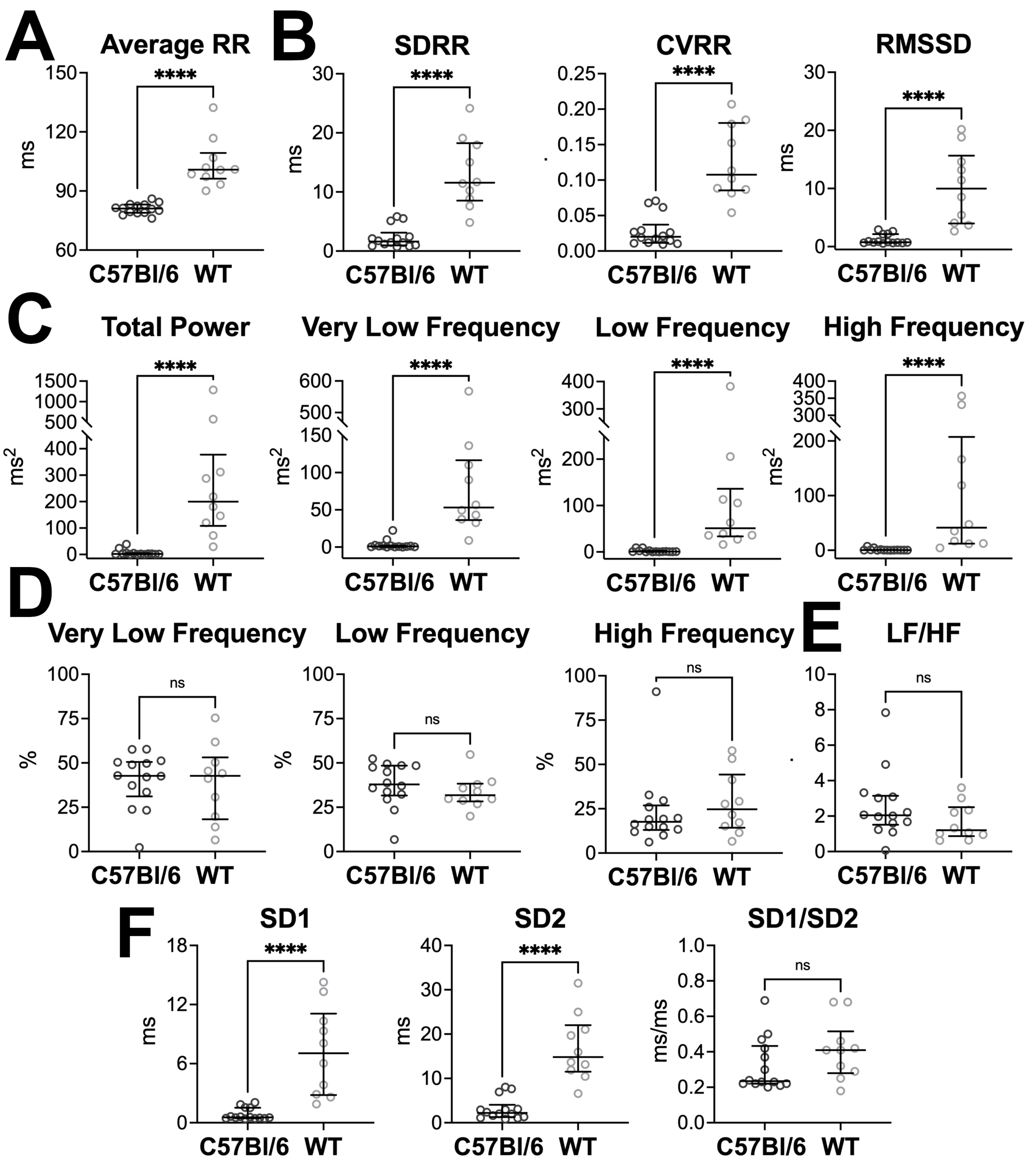

Supplementary Figure 2 | Heart rate dynamics in naïve female C57Bl/6 and WT mice. (A) Quantitative data for average RR interval duration. **(B)** Data for time-domain parameters of HRV. SDRR, standard deviation of RR intervals; CVRR, coefficient of variation of RR intervals; RMSSD, square root of the mean of the squared differences between adjacent RR. **(C)** Data for frequency-domain parameters of HRV. **(D)** Data for frequency bands normalized by total power. **(E)** Data for LF/HF ratio. **(F)** Data for nonlinear indices. Quantitative data were obtained from naïve C57Bl/6 (n = 14) and WT (n = 10) female mice. Data are shown as scattered plots with median and interquartile ranges. ns, not significant, ****P < 0.0001 using unpaired t-test or Mann-Whitney test.

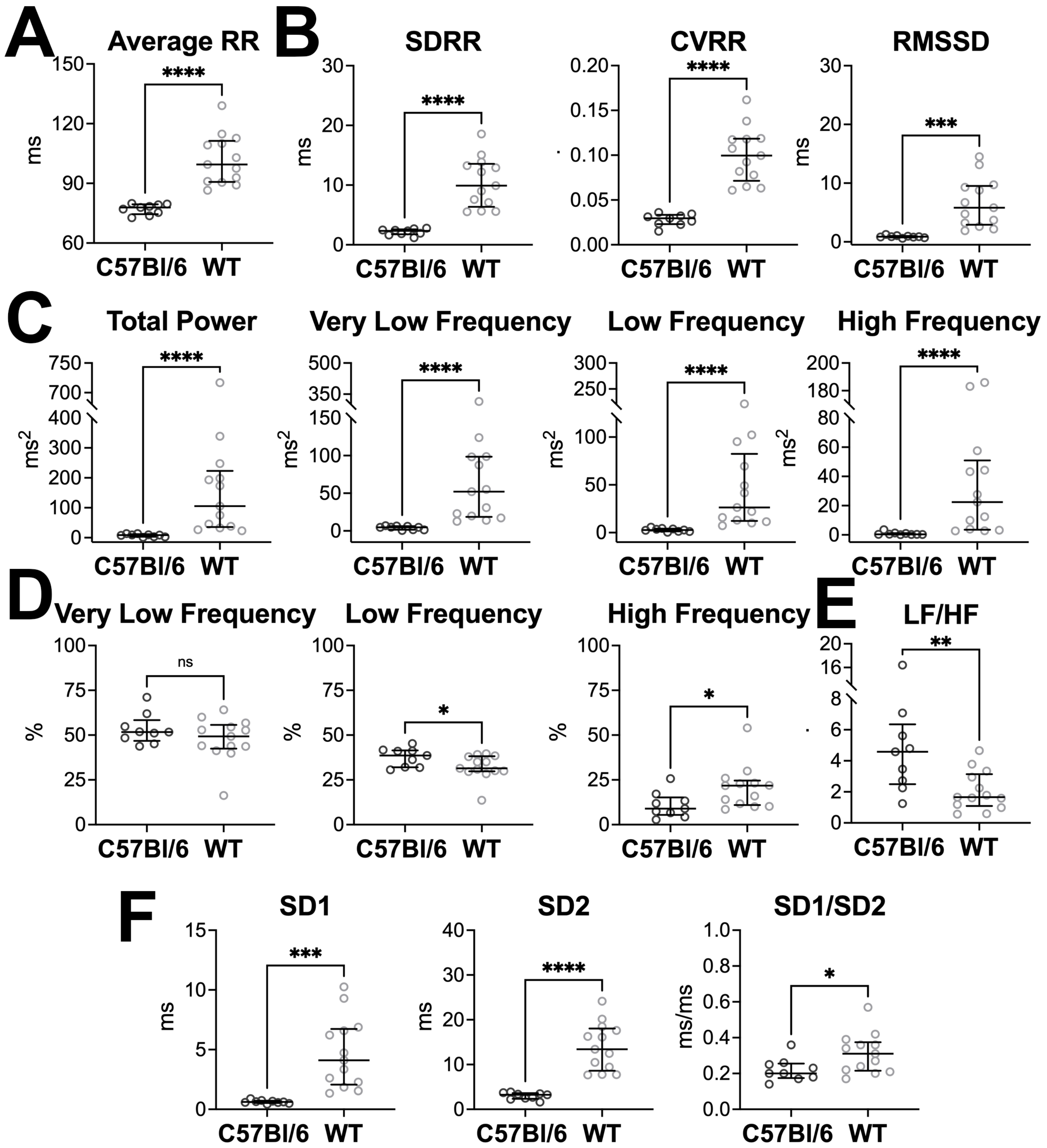

Supplementary Figure 3 | Heart rate dynamics in naïve male C57Bl/6 and WT mice. (A) Quantitative data for average RR interval duration. **(B)** Data for time-domain parameters of HRV. SDRR, standard deviation of RR intervals; CVRR, coefficient of variation of RR intervals; RMSSD, square root of the mean of the squared differences between adjacent RR. **(C)** Data for frequency-domain parameters of HRV. **(D)** Data for frequency bands normalized by total power. **(E)** Data for LF/HF ratio. **(F)** Data for nonlinear indices. Quantitative data were obtained from naïve C57Bl/6 (n = 9) and WT (n = 13) male mice. Data are shown as scattered plots with median and interquartile ranges. ns, not significant, *$P < 0.05$, **$P < 0.01$, ***$P < 0.001$, ****$P < 0.0001$ using unpaired *t*-test or Mann-Whitney test.

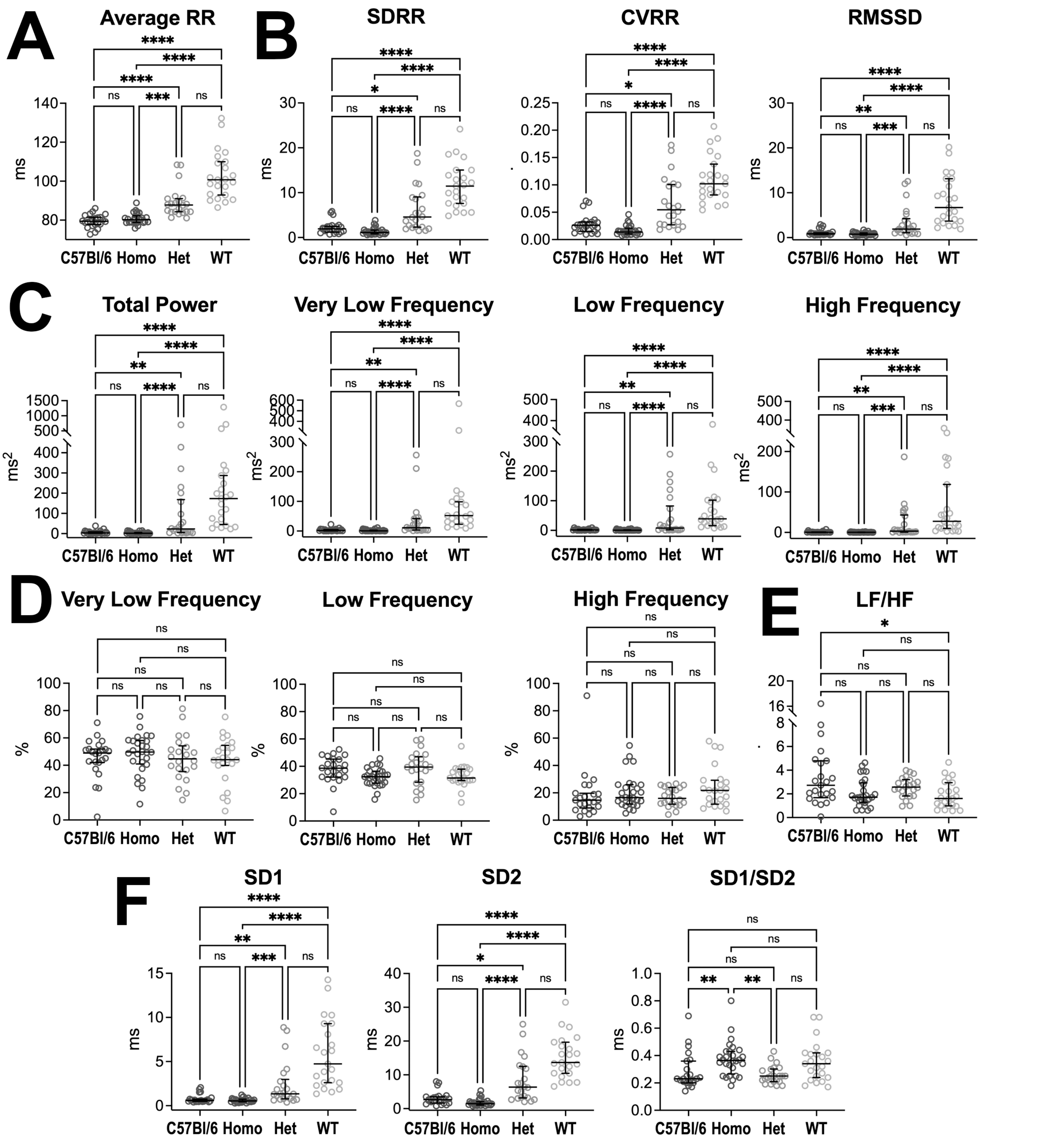

Supplementary Figure 4 | Heart rate dynamics in naïve male and female C57Bl/6, Homo, Het, and WT mice. **(A)** Quantitative data for average RR interval duration. **(B)** Data for time-domain parameters of HRV. SDRR, standard deviation of RR intervals; CVRR, coefficient of variation of RR intervals; RMSSD, square root of the mean of the squared differences between adjacent RR. **(C)** Data for frequency-domain parameters of HRV. **(D)** Data for frequency bands normalized by total power. **(E)** Data for LF/HF ratio. **(F)** Data for nonlinear indices. Quantitative data were obtained from naïve C57Bl/6 (n = 23), Homo (C57Bl/6 background, n = 28), Het (hybrid C57Bl/6 and 129/Sv background, n = 22), and WT (129/Sv background, n = 23) male and female mice. Data are shown as scattered plots with median and interquartile ranges. ns, not significant, $^{*}P < 0.05$, $^{**}P < 0.01$, $^{***}P < 0.001$, $^{****}P < 0.0001$ using Kruskal–Wallis one-way analysis of variance on ranks followed by Dunn's multiple comparison test.

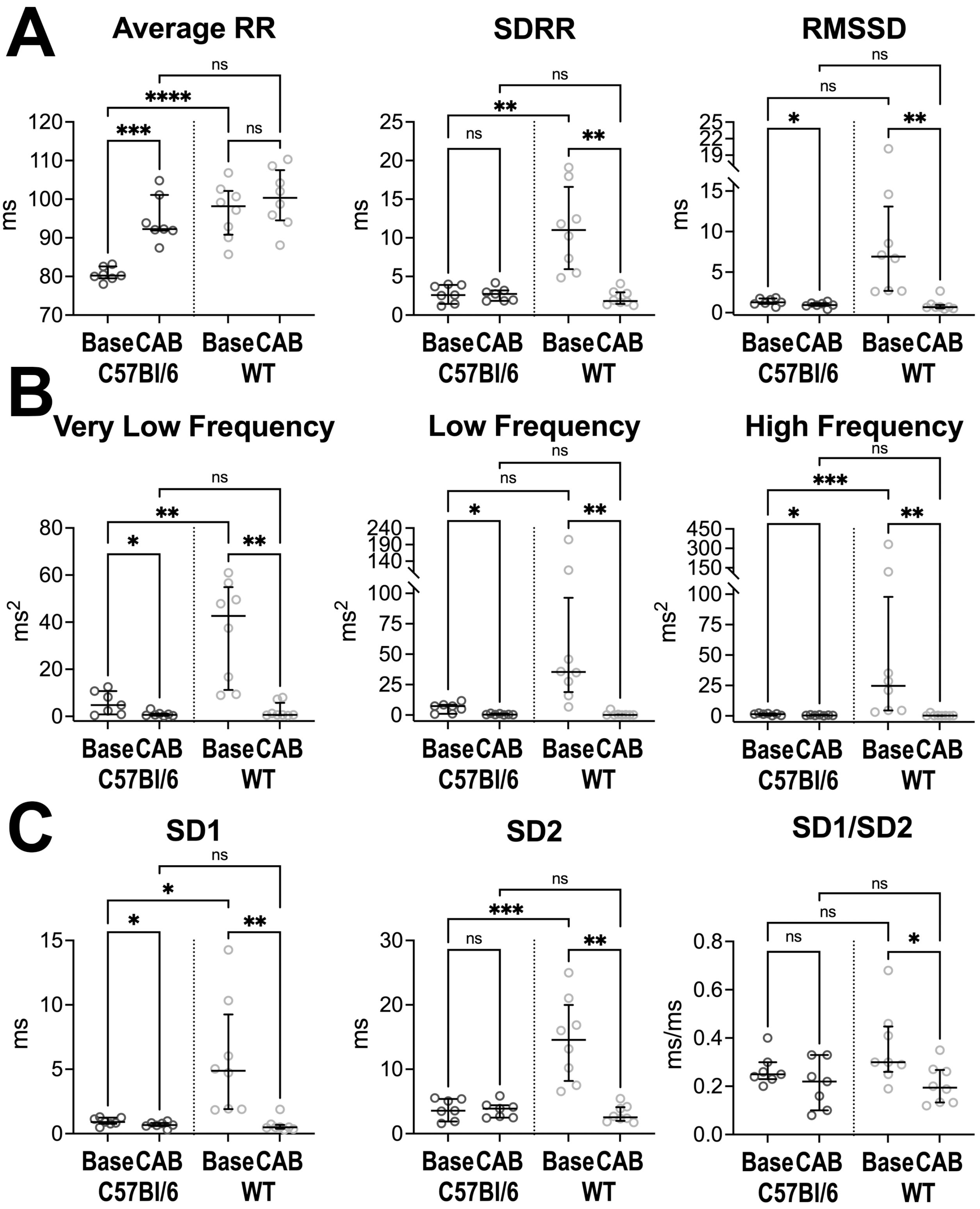

Supplementary Figure 5 | Inhibition of the autonomic nervous system and heart rate dynamics in naïve C57Bl/6 and WT female mice. (A) Quantitative data for RR interval duration and time-domain parameters of HRV for C57Bl/6 and WT mice before (baseline, Base) and after combined autonomic block with atropine and propranolol (CAB). **(B)** Data for frequency-domain parameters of HRV. **(C)** Data for nonlinear indices. Quantitative data were obtained from naïve C57Bl/6 (n = 7) and WT (n = 8) female mice. Data are shown as scattered plots with median and interquartile ranges. ns, not significant, **P* < 0.05, ***P* < 0.01, ****P* < 0.001, *****P* < 0.0001 using paired *t*-test or Wilcoxon signed rank test for comparisons within each genotype and unpaired *t*-test or Mann-Whitney test for comparisons across genotypes.

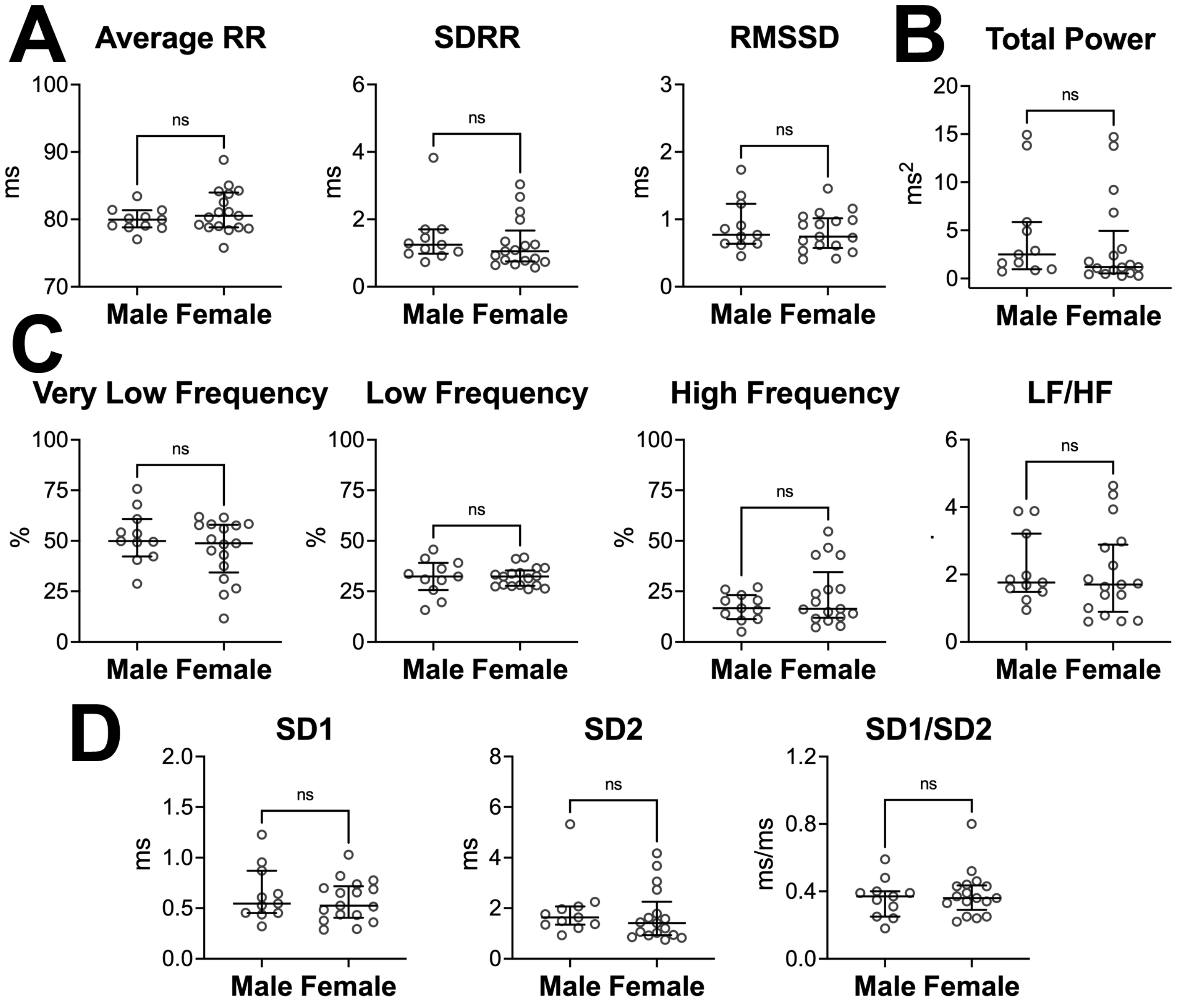

Supplementary Figure 6 | Heart rate dynamics in male and female naïve Homo mice. Quantitative data for average RR interval duration and time-domain parameters of HRV **(A)**, frequency-domain parameters **(B, C)**, and nonlinear indices **(D)** for male (n = 11) and female (n = 17) naïve Homo mice. Data are shown as scattered plots with median and interquartile ranges. ns, not significant.

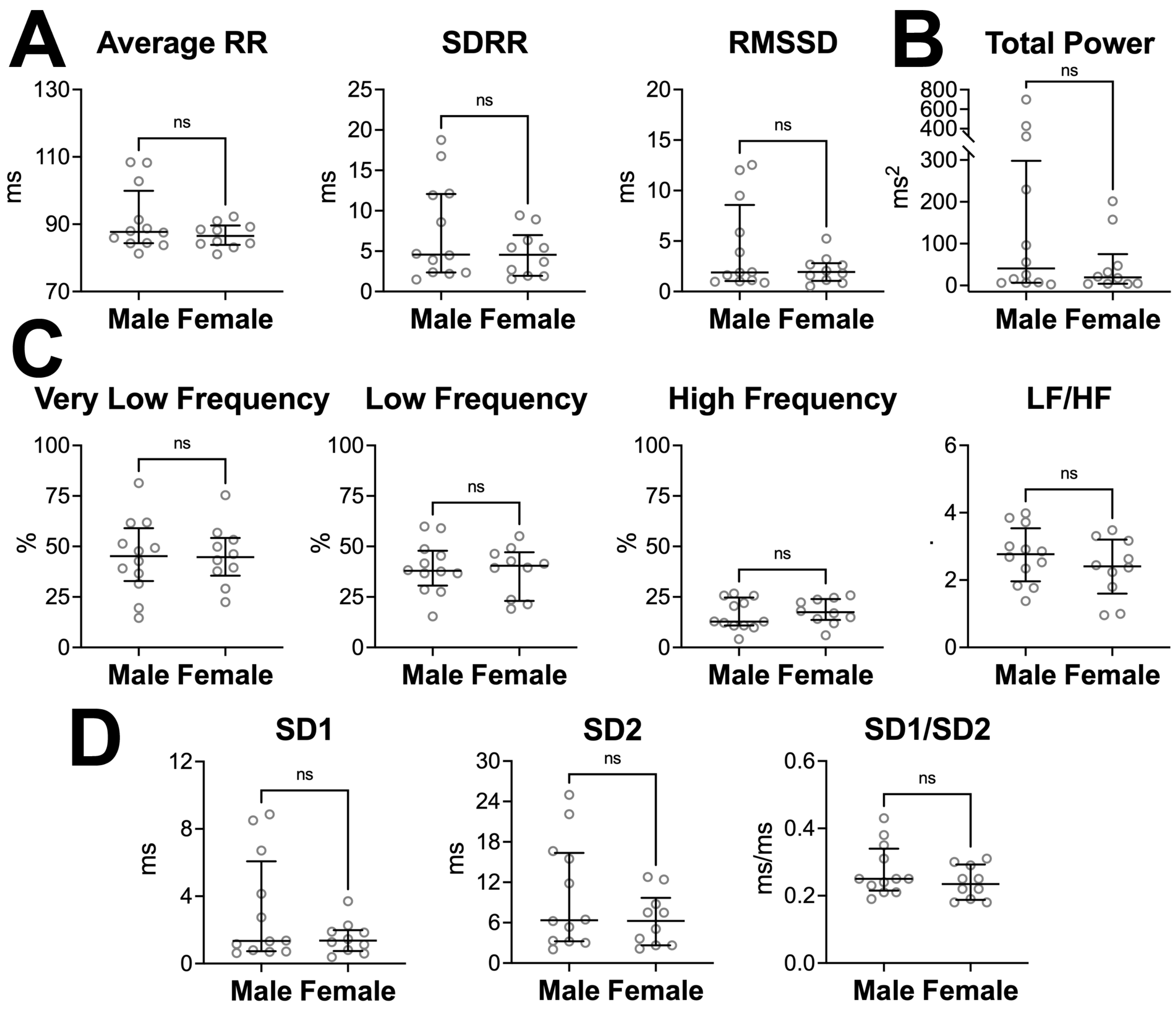

Supplementary Figure 7 | Heart rate dynamics in male and female naïve Het mice. Quantitative data for average RR interval duration and time-domain parameters of HRV **(A)**, frequency-domain parameters **(B, C)**, and nonlinear indices **(D)** for male (n = 12) and female (n = 10) naïve Het mice. Data are shown as scattered plots with median and interquartile ranges. ns, not significant.

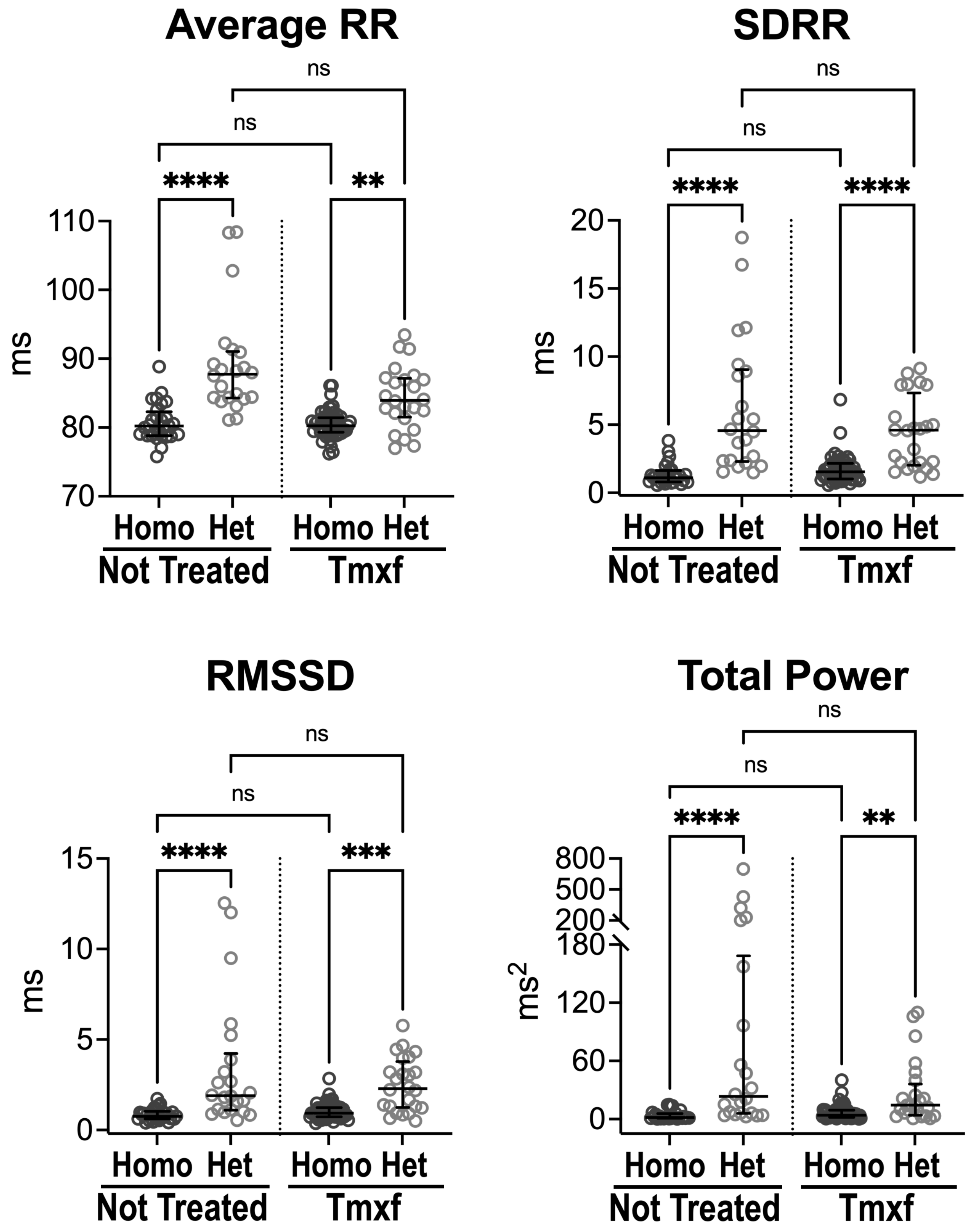

Supplementary Figure 8 | Heart rate dynamics and tamoxifen treatment in naïve mice. Quantitative data for average RR interval duration, time-domain parameters of HRV, and total power of RR interval variation for not treated (Not Treated) Homo (n = 28) and Het (n = 22) naïve mice of both sexes, and Homo tamoxifen (Tmxf)-treated (n = 53) and Het Tmxf-treated (n = 24) naïve mice of either sex. Data are shown as scattered plots with median and interquartile ranges. ns, not significant, $^{**}P < 0.01$, $^{***}P < 0.001$, $^{****}P < 0.0001$ using Kruskal–Wallis one-way analysis of variance on ranks followed by Dunn's multiple comparison test.

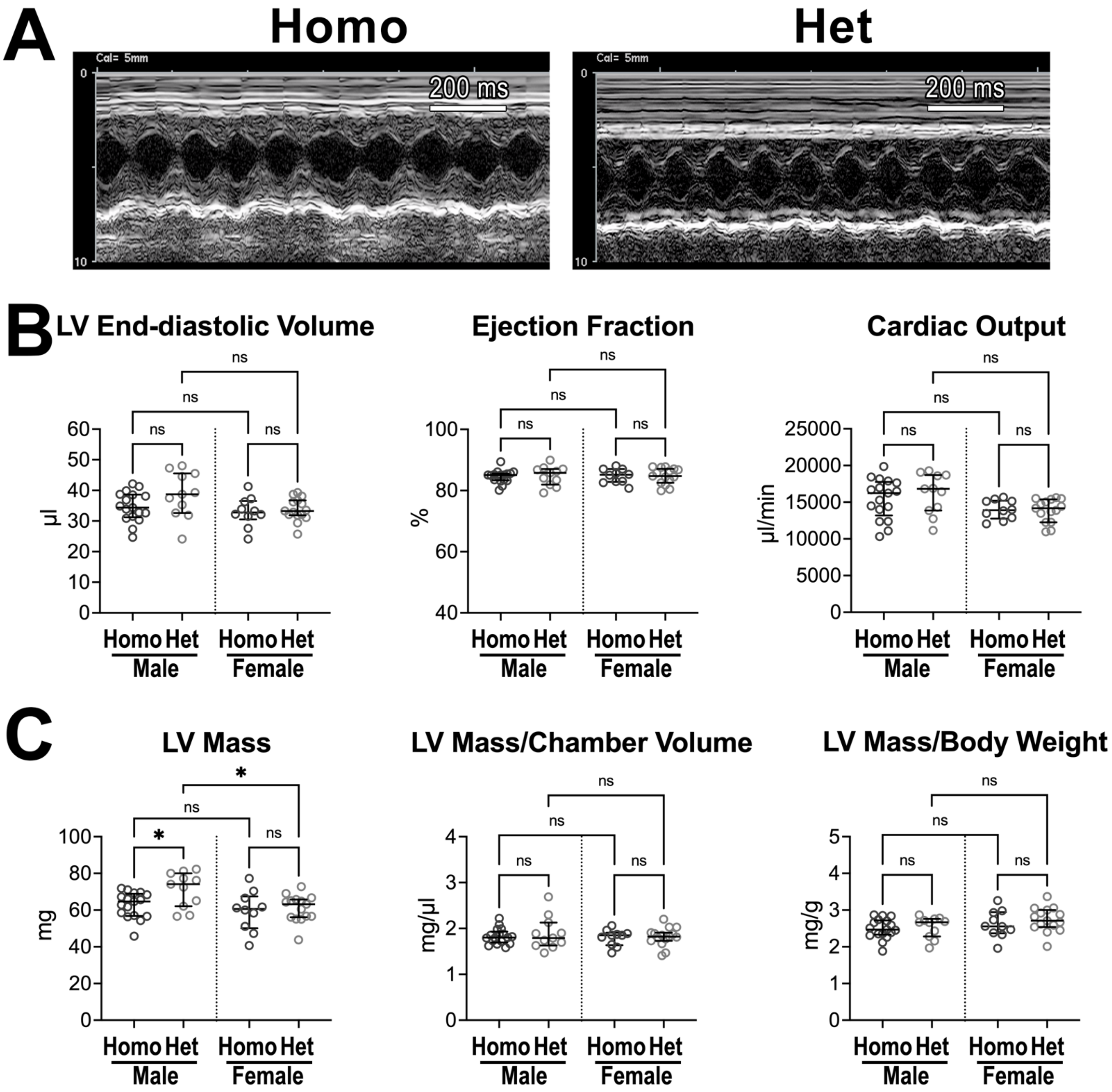

Supplementary Figure 9 | Cardiac function and anatomy in Homo and Het naïve mice. (A) M-mode echocardiographic images for Homo (left) and Het (right) male naïve mice. **(B, C)** Quantitative data for echocardiographic parameters obtained in male Homo (n = 17), male Het (n = 11), female Homo (n = 10), and female Het (n = 15) mice. Data are shown as scattered plots with median and interquartile ranges. ns, not significant, *P < 0.05 using one-way ANOVA followed by Bonferroni test.

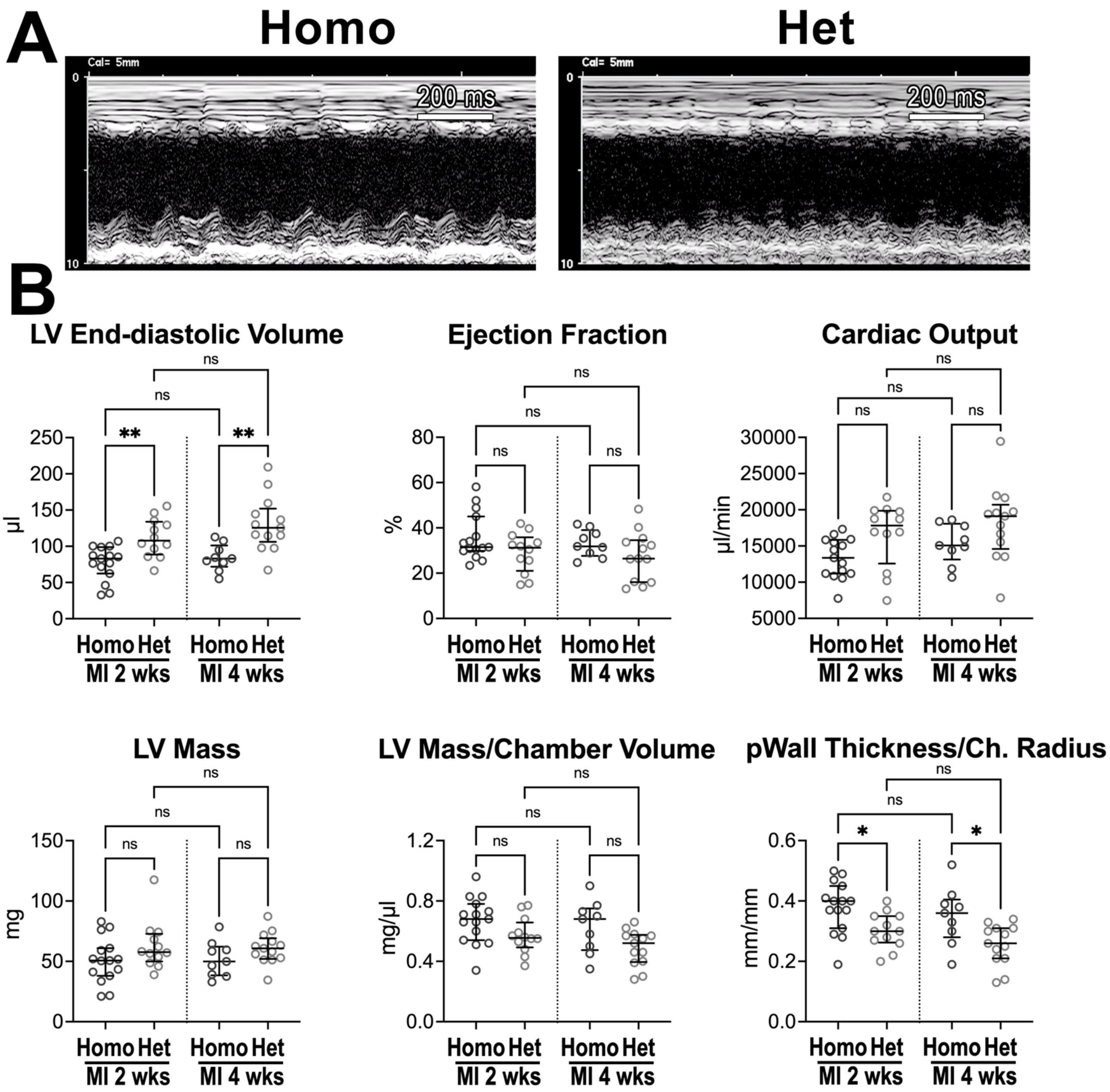

Supplementary Figure 10 | Cardiac function and anatomy in Homo and Het mice following myocardial infarction. (A) M-mode echocardiographic images for Homo (left) and Het (right) male mice at 14 days after myocardial infarction. **(B)** Quantitative data for echocardiographic parameters at 2 weeks after myocardial infarction (MI 2 wks) in Homo (n = 15; 7 males, 8 females) and Het (n = 12; 4 males, 8 females) mice and at 4 weeks after myocardial infarction (MI 4 wks) in Homo (n = 9; 8 males, 1 female) and Het (n = 13; 4 males, 9 females) mice. Male and female animals were combined. Data are shown as scattered plots with median and interquartile ranges. ns, not significant, *$P < 0.05$ using one-way ANOVA followed by Bonferroni test.

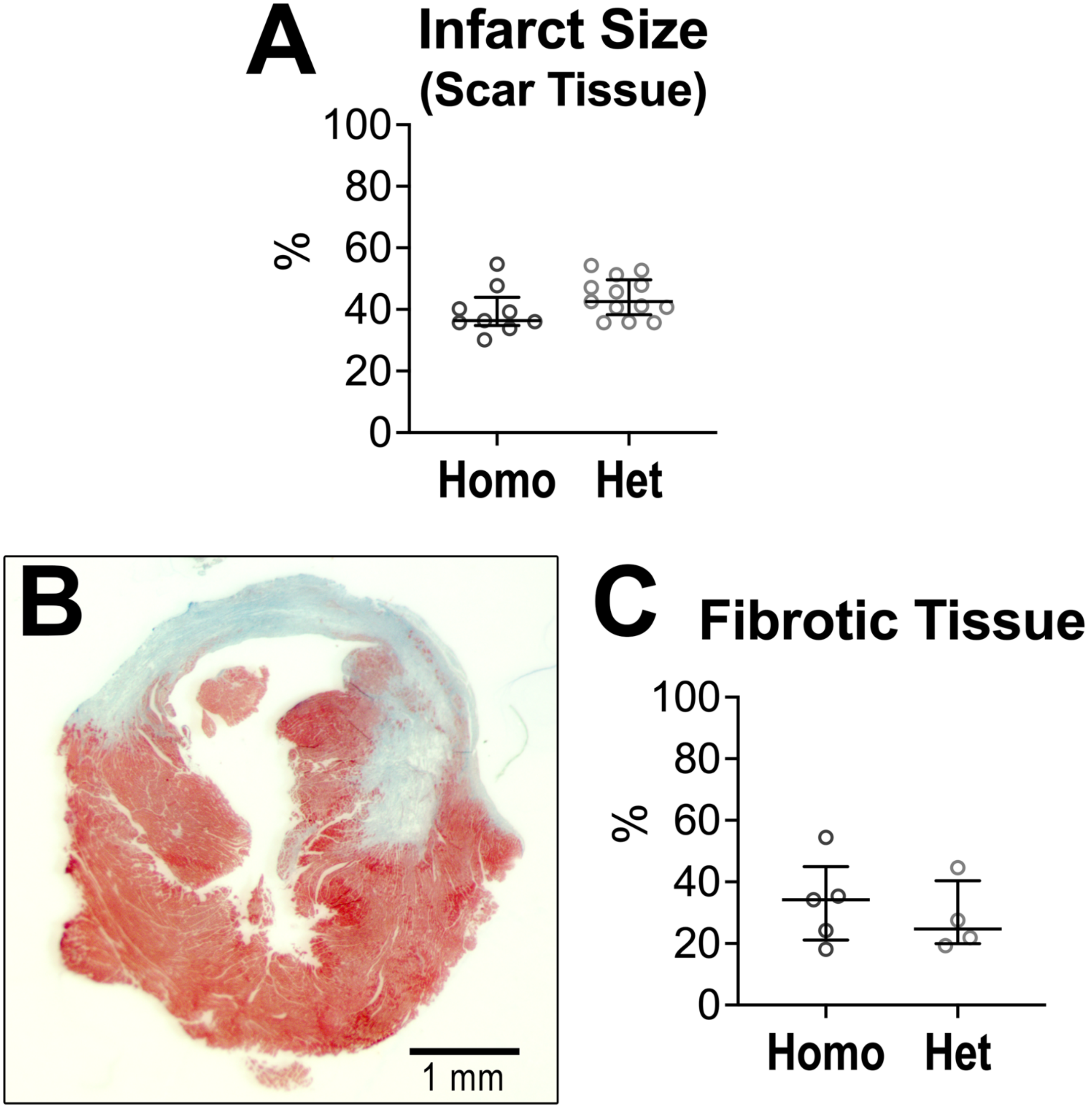

Supplementary Figure 11 | Infarct size in Homo and Het mice. (A) Quantitative data for infarct size evaluated in formalin-fixed transverse sections of the LV at the mid-ventricular level of Homo (n = 9) and Het (n = 13) mice, using the midline length approach. **(B)** Transverse section of the LV myocardium obtained from an infarcted Homo mouse. Fibrotic tissue is identified by trichrome staining. **(C)** Quantification of the area of fibrotic tissue with respect to the entire section for Homo (n = 5) and Het (n = 4) mice.

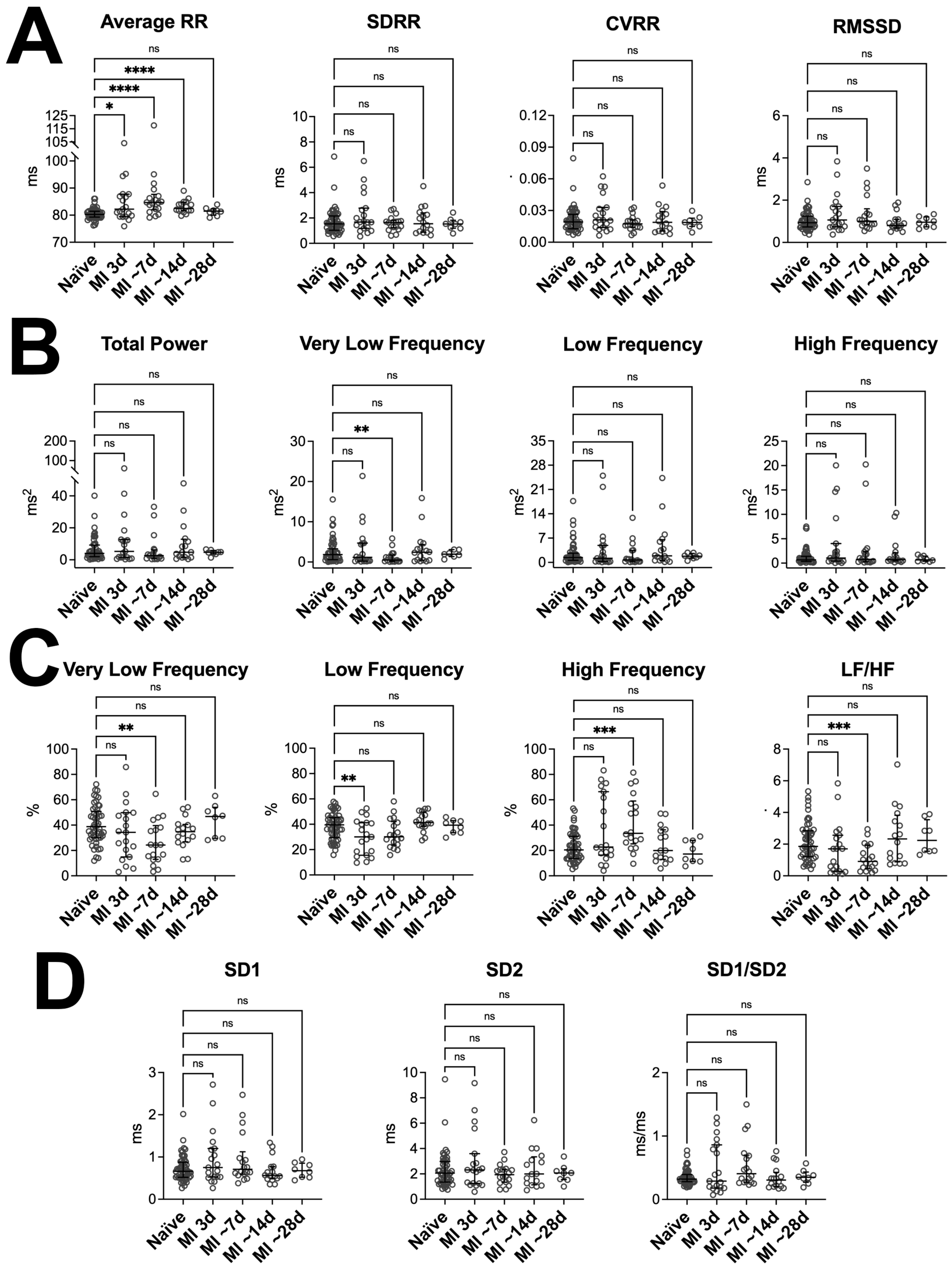

Supplementary Figure 12 | Heart rate dynamics in Homo mice following myocardial infarction. (A-D) Quantitative data for time-domain parameters **(A)**, frequency-domain parameters of HRV **(B)**, normalized frequency bands **(C)** and nonlinear indices **(D)**. Quantitative data were obtained from Homo naïve male and female mice (n = 53) and Homo male and female mice at 3 (n = 19), 7-9 (~7, n = 18)), 13-15 (~14, n = 16), and 28 (~28, n = 8) days after MI. ns, not significant, **P* < 0.05, ***P* < 0.01, ****P* < 0.001, *****P* < 0.0001 using unpaired *t*-test or Mann-Whitney test.

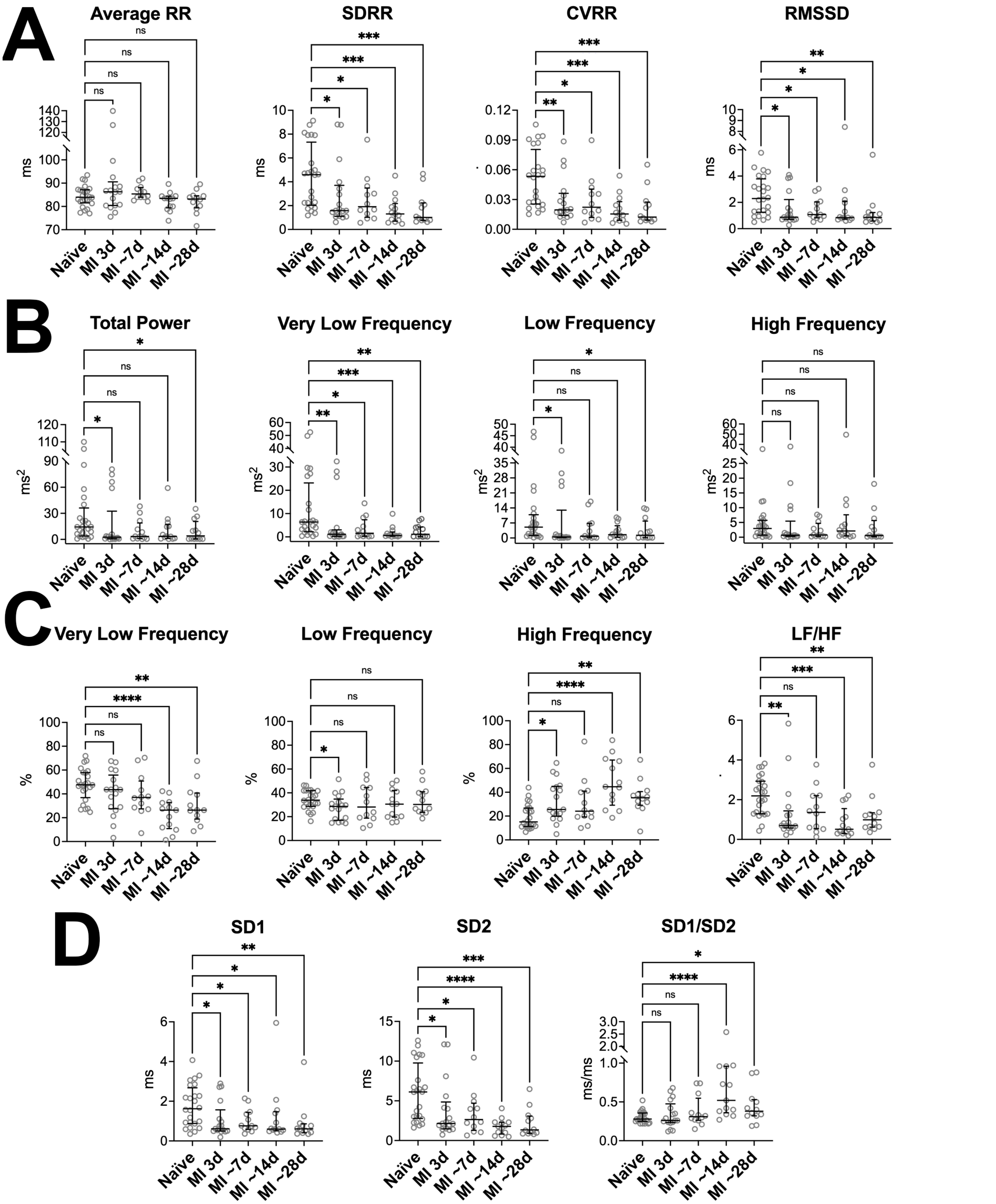

Supplementary Figure 13 | Heart rate dynamics in Het mice following myocardial infarction. (A-D) Quantitative data for time-domain parameters **(A)**, frequency-domain parameters of HRV **(B)**, normalized frequency bands **(C)** and nonlinear indices **(D)**. Quantitative data were obtained from Het naïve male and female mice (n = 24) and Het male and female mice at 3 (n = 17), 7-8 (~7, n = 12), 13-15 (~14, n = 13), and 29-33 (~28, n = 12) days after MI. Data are shown as scattered plots with median and interquartile ranges. ns, not significant, *$P < 0.05$, **$P < 0.01$, ***$P < 0.001$, ****$P < 0.0001$ using unpaired *t*-test or Mann-Whitney test.

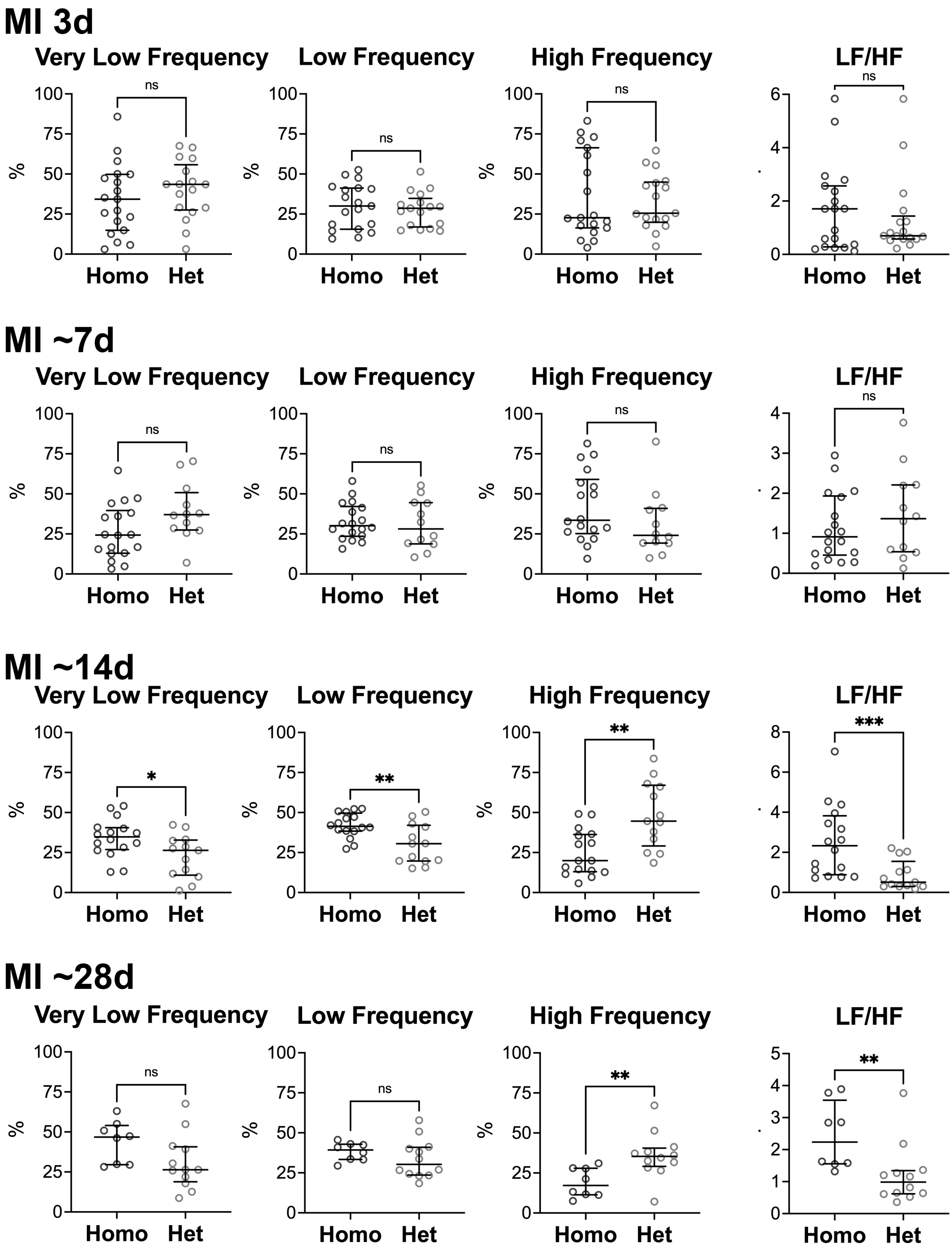

Supplementary Figure 14 | Heart rate dynamics in Homo and Het mice following myocardial infarction. Comparison of quantitative data for time-domain parameters and relative contribution of frequency bands in Homo and Het mice after myocardial infarction. Quantitative data correspond to data shown in Supplementary Figure 7 and Supplementary Figure 8. ns, not significant, *P < 0.05, **P < 0.01 using unpaired t-test.

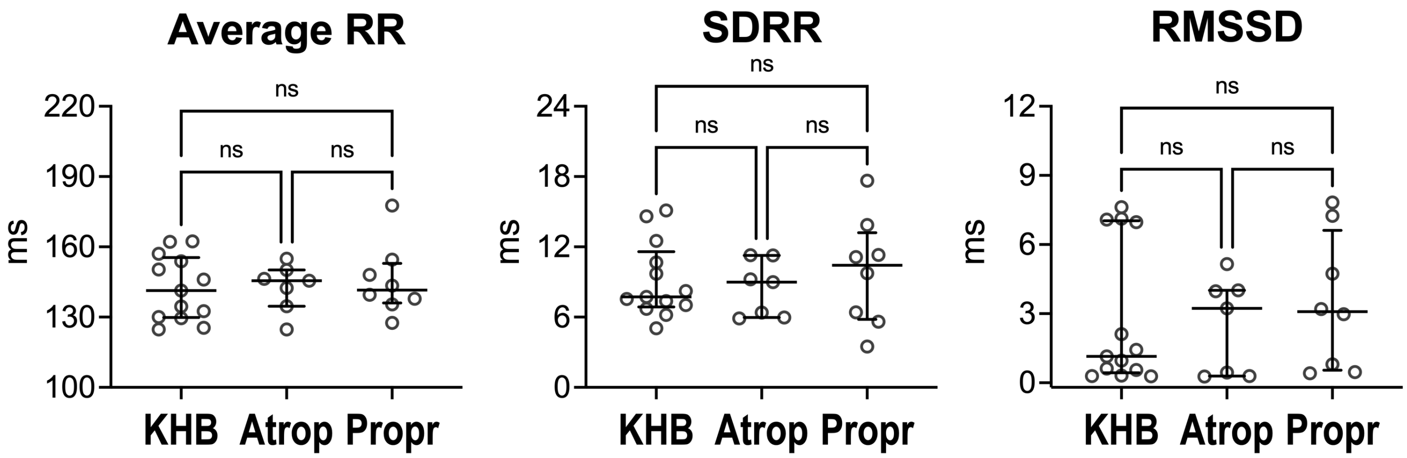

Supplementary Figure 15 | Intrinsic beating rate dynamics of the explanted, perfused heart. Quantitative data for average RR interval duration and time-domain parameters of HRV in naïve hearts from Homo male and female mice perfused with KHB buffer alone (n = 13), in the presence of atropine (Atrop, 100 nM, n = 7), or propranolol (Propr, 1 µM, n = 8). Data are shown as scattered plots with median and interquartile ranges. ns, not significant, using one-way ANOVA or Kruskal–Wallis one-way analysis of variance.

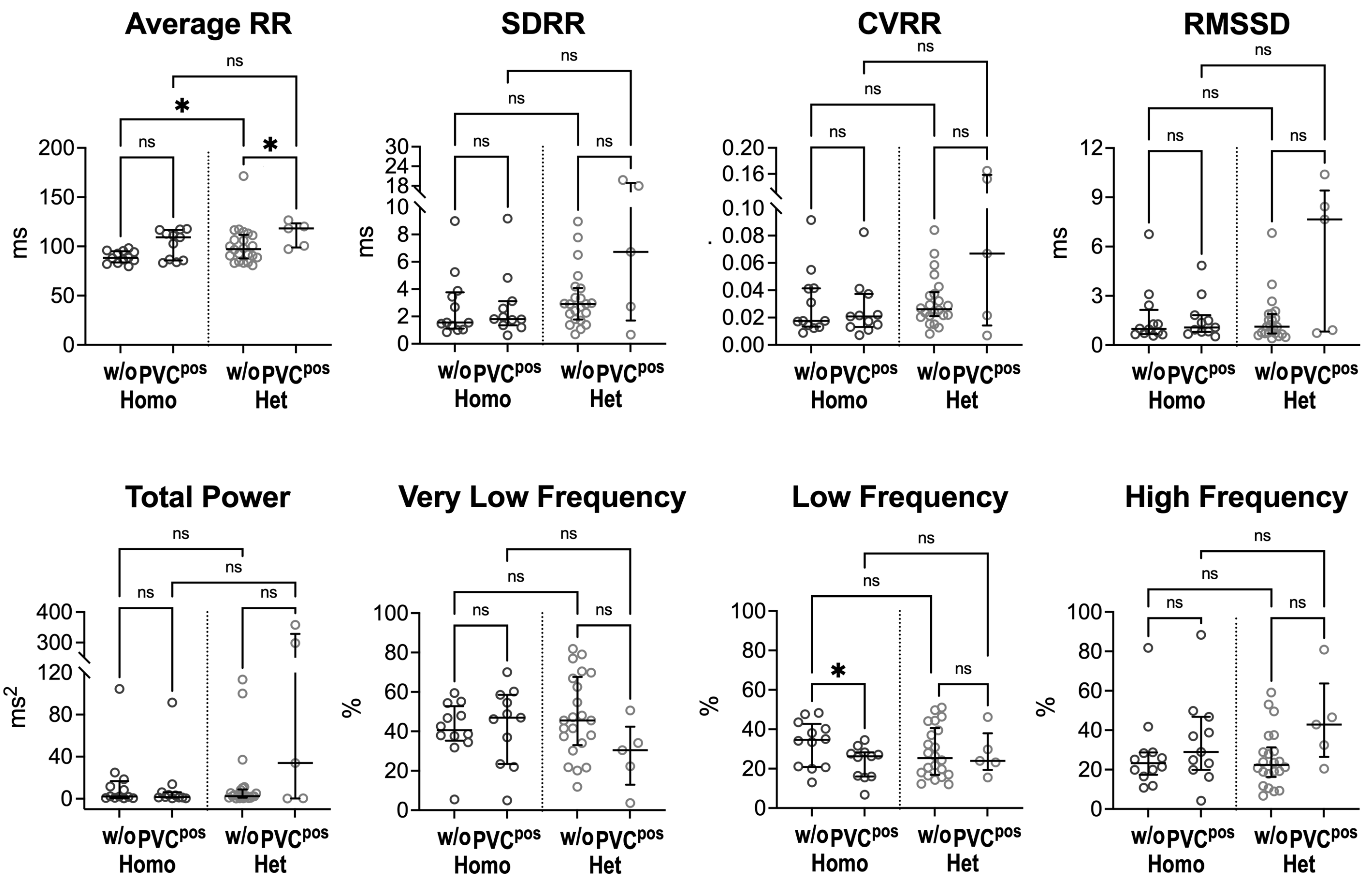

Supplementary Figure 16 | Heart rate dynamics in mice with and without recurrent ectopic events at 1 day after myocardial infarction. Comparison of quantitative data for time-domain parameters, total power, and relative contribution of frequency bands in Homo (n = 12, 11) and Het (n = 22, 5) mice in which recurrent PVCs were not observed (w/o) or detected (PVC^{pos}), at 1 day after myocardial infarction. Data are shown as scattered plots with median and interquartile ranges. ns, not significant, $^{*}P < 0.05$ using unpaired *t*-test or Mann-Whitney test.
